# Adrenal Aldosterone Synthase Expression Imaging in Primary Aldosteronism

**DOI:** 10.1056/NEJMc2507481

**Published:** 2025-11-27

**Authors:** Erik Årstad, Kerstin Sander, Tom R. Kurzawinski, Cameron Anderson, Yazead Buhidma, Ramla O. Awais, Fatih Sirindil, Robert Shortman, Louise Dickinson, Aidan G. O’Keeffe, John Dickson, Teng-Teng Chung, Morris J. Brown, Bryan Williams

**Affiliations:** 1 University College London, London; 2 University College London Hospitals NHS, London; 3 University of Nottingham, Nottingham, United Kingdom; 4 Queen Mary University of London, London

**To the Editor:**

Classical primary aldosteronism (PA) is the extreme phenotype of a widely prevalent syndrome of dysregulated aldosterone production involved in the pathogenesis of a substantial proportion of patients with hypertension, cardiovascular, metabolic and kidney disease.^1^ While bilateral PA requires lifelong medical therapy, adrenalectomy is recommended for patients with unilateral disease.^2^ Adrenal vein sampling (AVS), the current standard for guiding treatment decisions, is suboptimal, as it is technically complex, invasive and not often performed.^3^ Aiming to simplify and improve patient stratification, we developed an aldosterone synthase (CYP11B2)-specific ^18^F-labeled radioligand for imaging of PA pathology with positron emission tomography (PET).^4^

We herein report a pilot PET-CT study using our CYP11B2 specific radioligand in 17 consecutive patients with confirmed PA with lateralized findings on AVS (Table S1) who were scheduled for adrenalectomy (UK study registration ISRCTN58338025). The protocol and SAP are posted at NEJM.org. The demographics of the study population were broadly representative of patients with PA (Table S2). PET findings were validated by radioligand autoradiography and immunohistochemistry (IHC) on resected adrenal tissue. Details of methods, imaging results (Table S3 and Figs. S1-S17), and surgical outcomes (Table S4) for all participants are provided in the Supplementary Appendix, available at NEJM.org.

There were no adverse events reported with the radioligand. PET-CT showed avid uptake in adrenal lesions, with rapid clearance from adjacent and contralateral adrenal tissue and most other organs (Figs. 1 and S1). One or more lesions (total 22) were detected in all 17 adrenals predicted to be dominant by AVS. In five cases, microlesions (<1 cm^3^ by PET) were also detected in the contralateral adrenal (Figs. S1-S5). PET-CT showed several-fold higher standard uptake values (SUV range 1–13, median 6.5) in AVS-dominant versus contralateral adrenals (Table S3). Moreover, when accounting for lesion volume, the side of the dominant adrenal identified by our method was consistent with AVS in all 17 cases (Table S1). Importantly, CYP11B2 PET-CT not only detected macroscopic adrenal lesions but also revealed PA pathology in two patients with multiple aldosterone-producing micronodules (Figs. S5 and S6).^5^ Notably, almost half (12 out of 27) of the adrenal lesions detected with PET were not seen on CT or MRI reported independently by an experienced adrenal radiologist (Fig. S7). In contrast, 10 out of 25 discrete nodules detected with CT were negative on PET (Fig. S8). Serendipitously, two participants were found to have cortisol co-secretion, confirmed by both overnight dexamethasone suppression test (Table S1) and IHC, which enabled us to demonstrate that the radioligand binds exclusively to CYP11B2 (Fig. S9). We are now undertaking a Phase II trial to assess the performance of CYP11B2 PET-CT with our radioligand in a broader population of patients with PA. We speculate that this method may have the potential to alter treatment stratification for patients across the wide spectrum of aldosterone dysfunction, including classical PA as currently defined.^2^

In conclusion, this method of PET-CT appears to selectively detect CYP11B2 positive macro- and microscopic adrenal lesions.

## Supplementary Material

Supplementary Appendix

## Figures and Tables

**Figure 1:**
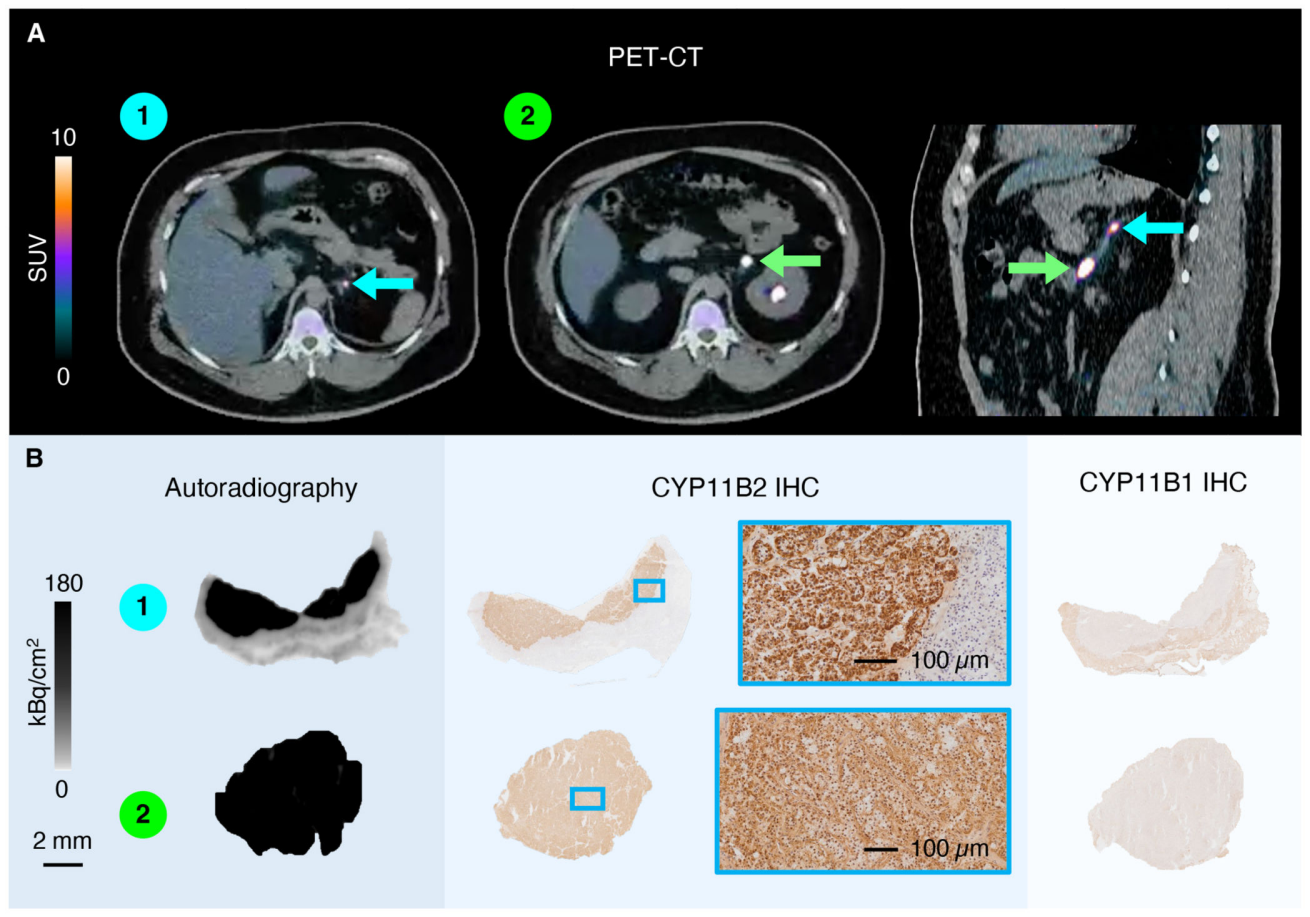
PET-CT and Tissue Imaging in Participant 17 **Panel A** shows PET overlaid on CT for participant 17 as a static scan from 35-45 min post-administration of the CYP11B2 specific radioligand, which was deemed optimal for diagnostic imaging. The images are displayed with an artificial color scale from 0-10 SUV. Two adrenal lesions were detected by PET-CT in the left adrenal, which was predicted to be dominant by AVS. The left image shows an axial view of the superior microlesion. The middle image shows an axial view of the inferior macroscopic lesion. Excretion of the radiotracer in the renal pelvis can be seen. The right image displays a sagittal view of both adrenal lesions. There was no detectable PET signal in the contralateral adrenal gland or in adrenal tissue surrounding the lesions. **Panel B** shows radioligand autoradiography (left), and immunohistochemical staining of aldosterone synthase (CYP11B2, middle) and the cortisol-producing enzyme 11β-hydroxylase (CYP11B1, right) performed on directly adjacent sections of the resected adrenal tissue. Radioligand binding was quantified using internal standards in each experiment, and images are displayed on a grey scale representing the tracer binding between 0 and 180 kBq/cm^2^. The AVS lateralization index was 17.1 with ACTH stimulation. The overnight dexamethasone suppression cortisol was 44 nmol/l. At 6 and 12 months after adrenalectomy, the patient achieved complete biochemical success and partial clinical response according to PASO criteria.
