# Supplementary material for: Adrenal Aldosterone Synthase Expression Imaging in Primary Aldosteronism
Source: N Engl J Med. Author manuscript; Available in PMC 2025 Dec 3. (PMC7618428; doi:10.1056/NEJMc2507481)
Supplement: Supplementary Appendix [file EMS210300-supplement-Supplementary_Appendix.pdf]

# Adrenal Aldosterone Synthase Expression Imaging in Primary Aldosteronism

## TABLE OF CONTENTS 1


**LIST OF INVESTIGATORS 3**

INVESTIGATORS 3

CONFLICTS OF INTEREST 4

STUDY TEAM 5

RECRUITING HOSPITALS 5

RESEARCH SITES 6

ACKNOWLEDGEMENTS 6

**METHODS 7**

STUDY DESIGN 7

MANUFACTURING OF THE RADIOPHARMACEUTICAL INJECTION 7

PATIENT CHARACTERISTICS 8

PET-CT IMAGING 9

COLLECTION AND PROCESSING OF SURGICAL SPECIMENS 9

AUTORADIOGRAPHY 10

IMMUNOHISTOCHEMICAL STAINING AND HISTOLOGY 10

**SUPPLEMENTARY TABLES 12**

TABLE S1. CORTISOL CO-SECRETION, AVS AND PET RESULTS 12

TABLE S2. REPRESENTATIVENESS OF STUDY PARTICIPANTS 14

TABLE S3. PET-CT FINDINGS 16

TABLE S4. BIOCHEMICAL AND CLINICAL RESPONSE TO ADRENALECTOMY 18

**SUPPLEMENTARY FIGURES 20**

FIGURE S1. DISTRIBUTION OF RADIOLIGAND UPTAKE OVER TIME 20

FIGURE S2. PARTICIPANT 06 22
FIGURE S3. PARTICIPANT 07 23
FIGURE S3. PARTICIPANT 07, *CONTINUED* 25
FIGURE S4. PARTICIPANT 08 26
FIGURE S5. PARTICIPANT 12 29
FIGURE S5. PARTICIPANT 12, *CONTINUED* 29
FIGURE S6. PARTICIPANT 14 30
FIGURE S7. ADRENAL LESIONS DETECTED BY PET THAT WERE NOT SEEN ON CT 32
FIGURE S8. ABSENCE OF RADIOTRACER UPTAKE IN CT POSITIVE NODULES 33
FIGURE S9. RADIOLIGAND BINDING IN ADRENAL TISSUE FROM PARTICIPANTS WITH CORTISOL CO-SECRETION 34
FIGURE S10. PARTICIPANT 03 36
FIGURE S11. PARTICIPANT 05 38
FIGURE S12. PARTICIPANT 09 38
FIGURE S13. PARTICIPANT 10 39
FIGURE S14. PARTICIPANT 11 41
FIGURE S15. PARTICIPANT 13 41
FIGURE S16. PARTICIPANT 15 42
FIGURE S17. PARTICIPANT 16 43

REFERENCES 45

## LIST OF INVESTIGATORS

INVESTIGATORS

| | |
|---|---|
| Cameron **Anderson** | Nuclear Medicine Physicist, University College London Hospitals NHS Foundation Trust, Institute of Nuclear Medicine, London, UK |
| Erik **Årstad** | Professor in Radiochemistry, University College London, Centre for Radiopharmaceutical Chemistry, London, UK |
| Ramla O **Awais** | Head of Quality Assurance (Qualified Person), University College London, Centre for Radiopharmaceutical Chemistry, London, UK |
| Morris J **Brown** | Professor of Endocrine Hypertension, Queen Mary University of London, William Harvey Research Institute, Centre for Clinical Pharmacology and Precision Medicine, London, UK |
| Yazead **Buhidma** | Research Fellow, University College London, Centre for Radiopharmaceutical Chemistry, London, UK |
| Teng-Teng **Chung** | Consultant Endocrinologist, University College London Hospitals NHS Foundation Trust, Department of Endocrinology, London, UK |
| Louise **Dickinson** | Consultant Radiologist, University College London Hospitals NHS Foundation Trust, Department of Radiology, London, UK |
| John **Dickson** | Head of Nuclear Medicine Physics, University College London Hospitals NHS Foundation Trust, Institute of Nuclear Medicine, London, UK |
| Tom R **Kurzawinski** | Consultant Endocrine Surgeon, University College London Hospitals NHS Foundation Trust, Department of Endocrine Surgery, London, UK |
| Aidan G **O'Keefe** | Associate Professor, University of Nottingham, School of Mathematical Sciences, Nottingham, UK |
| Kerstin **Sander** | Associate Professor, University College London, Centre for Radiopharmaceutical Chemistry, London, UK |
| Robert **Shortman** | Research Manager Nuclear Medicine, University College London Hospitals NHS Foundation Trust, Institute of Nuclear Medicine, London, UK |

| | |
|---|---|
| Fatih **Sirindil** | Research Fellow, University College London, Centre for Radiopharmaceutical Chemistry, London, UK; now Research Scientist, Eurofins Selcia, Ongar, UK |
| Bryan **Williams** | Chair of Medicine, University College London, Consultant Physician, University College London Hospitals NHS Foundation Trust, and Chief Scientific and Medical Officer, British Heart Foundation, London, UK |

CONFLICTS OF INTEREST

| | |
|---|---|
| Erik **Årstad** | Inventor on UCL Business owned patent patent (WO 2014/057291 'Compounds and their synthesis) of the fluorine-18 labelling chemistry that was used to manufacture [$^{18}$F]AldoView |
| Morris J **Brown** | Author of a number of publications about alternative PET-CT scans, as listed among the references; senior author of publications regarding other aldosterone synthase inhibitors, past or current member of Scientific Advisory Boards of companies developing these drugs, specifically Cincor and Astra-Zeneca |
| Kerstin **Sander** | Inventor on UCL Business owned patent patent (WO 2014/057291 'Compounds and their synthesis) of the fluorine-18 labelling chemistry that was used to manufacture [$^{18}$F]AldoView |
| Bryan **Williams** | Chair of the steering committee for an Astra Zeneca sponsored Phase 3 clinical trial of an aldosterone synthase inhibitor, and member of steering committee/advisory board for novel antihypertensive drugs in development for Novartis, Roche, Alnylam and Antlia |

STUDY TEAM

| | |
|---|---|
| Tarek E **Abdel-Aziz** | Consultant Endocrine Surgeon, University College London Hospitals NHS Foundation Trust, Department of Endocrine Surgery, London, UK |
| Esra **Edaan** | Quality Control Chemist, University College London, Centre for Radiopharmaceutical Chemistry, London, UK |
| Alex **Freeman** | Consultant Histopathologist, University College London Hospitals NHS Foundation Trust, Department for Cellular Pathology and Histopathology, London, UK |
| Jennifer **Furman** | Senior Translational Research Manager, University College London, Translational Research Office, London, UK |
| Matthias **Glaser** | Senior Radiochemist, University College London, Centre for Radiopharmaceutical Chemistry, London, UK |
| Bernard **Khoo** | Consultant Endocrinologist, Royal Free London NHS Foundation Trust, London, UK |
| Radu **Mihai** | Consultant Endocrine Surgeon, Oxford University Hospitals NHS Trust, Oxford, UK |
| Dylan **Pritchard** | Radiochemist, University College London, Centre for Radiopharmaceutical Chemistry, London, UK |
| Nemanja **Stojanovic** | Consultant Physician in Endocrinology, Queen's Hospital, Romford, UK |
| Frazer **Twyman** | Head of Production, University College London, Centre for Radiopharmaceutical Chemistry, London, UK |
| Simon **Wan** | Radiologist, University College London Hospitals NHS Foundation Trust, Institute of Nuclear Medicine, London, UK |
| Lucinda **Winter** | Head of School for Pathology, Oxford University Hospitals NHS Foundation Trust, Oxford, UK |

RECRUITING HOSPITALS

University College London Hospitals NHS Foundation Trust

The London Clinic

Royal Free London NHS Foundation Trust

Oxford University Hospitals NHS Foundation Trust

Barts Health NHS Trust

## RESEARCH SITES

| | |
|---|---|
| Radiochemistry | University College London, Centre for Radiopharmaceutical Chemistry, GMP Facility |
| PET-CT | University College London Hospitals NHS Foundation Trust, Institute of Nuclear Medicine |
| Tissue imaging | University College London, Centre for Radiopharmaceutical Chemistry; University College London, Queen Square Institute of Neurology, Department of Neurodegenerative Diseases |
| Statistical analysis | University of Nottingham, School of Mathematical Sciences |

## ACKNOWLEDGEMENTS

We thank the patients and their families for participating in the IDEAL study. PET scanning was supported by the team at UCLH Institute of Nuclear Medicine, including Marie Meagher, Joshua Mc Andrew, and Raymond Endozo.

The study was funded by the Medical Research Council (MR/T005769/1) and the National Institute for Health and Care Research University College London Hospitals Biomedical Research Centre (BRC671/CV/ED/101320; BRC540/HEI/EA/110410). We acknowledge non-financial support from Merck Sharp & Dohme (MSD), which developed the CYP11B2-specific molecule that was radiolabeled to produce our radioligand.

Perplexity AI Sonar (Meta Llama 3.3 70B, September 2025 version) was used to assist with English-language editing during manuscript preparation.

# METHODS

## STUDY DESIGN

Image-Derived Enzymatic Adrenal Lateralization (IDEAL) was a pilot study. Participants were recruited for a single PET-CT scan at University College London Hospital. The protocol had a single inclusion criterion; 'patients identified as surgical candidates for removal of an Aldosterone Producing Adenoma'. Recruitment was based on Endocrine Society Clinical Practice guideline for the diagnosis of primary aldosteronism (PA) and suitability for surgery. Participants were enrolled consecutively based on the clinical indication for adrenalectomy. The absence of an adrenal nodule on cross-sectional imaging, or any other clinical characteristic, did not constitute exclusion criteria. All adrenal vein sampling (AVS) were performed under adrenocorticotropic hormone (ACTH) stimulation. Successful cannulation was defined by selectivity index, the ratio of adrenal vein cortisol to inferior vena cava of ≥3. Lateralization was determined using lateralization index (LI) calculated as the ratio of aldosterone to cortisol in the dominant adrenal vein to that in the non-dominant vein, with a threshold of ≥4, along with evidence of suppression of aldosterone-to-cortisol ratio in the non-dominant vein relative to the inferior vena cava.[1] All AVS lateralized to one side, and all patients had adrenalectomy.

Prior to their scheduled adrenalectomy, participants underwent PET-CT scanning with a radioligand developed to bind to aldosterone synthase (CYP11B2) with unprecedented selectivity.[2] All participants provided written informed consent. Patients remained on their routine anti-hypertensive medication regimen and underwent PET-CT imaging without prior dexamethasone suppression. Imaging results for all participants are included in this supplementary appendix apart from participant 17, whose results are presented in the main figure.

The study was conducted in accordance with the principles of the Declaration of Helsinki. The clinical study was approved by Brent Research Ethics Committee, UK (ref: 21/LO/0521) and tissue imaging was approved by Solihull Research Ethics Committee, UK (ref: 22/WM/0010). The study was registered (UK study registration ISRCTN58338025) on 22$^{nd}$ July 2022, and the first patient was enrolled on 11$^{th}$ August 2022.

## MANUFACTURING OF THE RADIOPHARMACEUTICAL INJECTION

The parent compound of the radioligand, cyclopropyl-5,6-difluoro-2-(5-fluoropyridin-3-yl)-1*H*-benzo[*d*]imidazole, is a small molecule, high affinity, highly selective aldosterone synthase inhibitor.[3] We have previously reported the

radiosynthetic route and preclinical characterization of the radioligand.[2] For this study, the radiosynthesis was automated at the University College London Good Manufacturing Practice Facility. The radioligand was obtained with an activity yield of 28 ±8% and a molar activity of 687 ±236 GBq/μmol (*n*=21) with a synthesis time of 1 hour.

## PATIENT CHARACTERISTICS

Seventeen participants with PA were included in the study (**Table S2**). The cohort exhibited the anticipated age at presentation and sex distribution.[4–6] The ethnic composition (participants self-identified as White, Asian or Black) reflects local diversity[7] but may underrepresent the proportion of Black patients described in global epidemiological studies of PA.[8] Confirmatory testing with saline infusion or captopril suppression was performed in 4 of 17 participants where clinically indicated. Overnight dexamethasone suppression testing was performed in 14 of 17 participants (**Table S1**). Cortisol levels suppressed to less than 50 nmol/L in all but two cases. Participants 02 and 04 showed biochemical evidence for cortisol co-secretion, with post-dexamethasone cortisol concentrations of 65 nmol/L and 58 nmol/L, respectively.

AVS lateralized excess aldosterone production to the right side in 11 participants and the left side in 6 participants. In 3 participants with failed right adrenal vein cannulation, the median aldosterone-to-cortisol ratio between the left adrenal vein and the inferior vena cava was 0.13 (range 0.03–0.30), below the normal threshold of 0.5. Suppression of the aldosterone-to-cortisol ratio in the left adrenal vein relative to the inferior vena cava supports lateralization of aldosterone excess to the right adrenal gland. 15 participants had adrenal nodules detected by CT or MR imaging in the adrenal predicted to be dominant by AVS.

One participant (11) had a lateralization index of 3.9 (cut-off = 4). Surgery was undertaken as a clinical decision because of unacceptable side effects from therapy with mineralocorticoid receptor antagonists (MRA). The decision was made with informed consent, with the aim of reducing the disease burden while acknowledging the potential risk of residual contralateral disease.

All participants showed a biochemical and/or clinical response, which was monitored for up to one year after adrenalectomy (**Table S4**).

### PET-CT IMAGING

All participants received 200 MBq (range 177–243 MBq, mean 205 MBq) of the radioligand as an intravenous bolus. Static PET-CT images were taken for 10 minutes, 35 minutes after injection, on a Siemens Biograph Vision 600 (Siemens Healthineers, Knoxville TN, USA) following standard CT for PET localization and attenuation correction (with Care kV reference: 120 kVp, CAREdose 4D (reference: 80 mAs), 3 mm slice thickness, and pitch 1 with reconstructed pixel size of 0.98 x 0.98 mm). PET images used for quantification were reconstructed in compliance with EANM/EARL guidelines Standard 2 (Ordered Subset Expectation Maximization (OSEM) with Time of Flight (TOF) information, 4 iterations, 5 subsets, 220 x 220 matrix, 3 mm slice thickness and a 3.5 mm gaussian filter).[9,10] PET images used for visual interpretation and display in this manuscript were reconstructed with OSEM with TOF and Point Spread Function (PSF) information, 4 iterations, 5 subsets, 440 x 440 matrix, 3 mm slice thickness and no filter. Fused PET-CT images were created in HorosTM with PET images (SUV scale between 0 and 10 with 'GE color' look up table (LUT)) overlaid on CT images. PET images were quantified using syngo.via VB80E (Siemens Healthineers, Erlangen, Germany). Automatic thresholding with a threshold of SUV = 4 was employed to create adrenal lesion volumes of interest (VOIs) for measurement of $SUV_{max}$, $SUV_{mean}$ and VOI volume. Radioligand uptake was calculated as adrenal lesion $SUV_{mean}$ x VOI volume and is expressed in arbitrary units (AU). Adrenals with no PET pixel above an SUV of 4 had their $SUV_{max}$ determined as the maximum pixel value within a spherical VOI placed over the adrenal as identified on CT.

### COLLECTION AND PROCESSING OF SURGICAL SPECIMENS

Adrenal surgical specimens were obtained from all participants, after written informed consent for research use. Ethical approval was granted by the Solihull Research Ethics Committee, UK (ref: 22/WM/0010).

For autoradiography and initial immunohistochemical staining (IHC), samples were immediately flash frozen in a slurry of isopentane and dry ice. Formalin fixed and paraffin embedded (FFPE) tissue blocks were used for further characterization, if required. Throughout the manuscript, FFPE tissue is referred to as 'fixed' tissue.

Frozen tissue samples were cryosectioned at 10 μm using a Bright Instruments Cryostat (OTF6000), and sections were mounted on SuperFrost Plus slides. Remaining tissue and slides were stored at -80°C until used for autoradiography and IHC staining.

### AUTORADIOGRAPHY

Thawed tissue sections on microscope slides were dipped in pentane for 5 seconds to degrease the tissue, rehydrated in tris-buffered saline (TBS; pH = 7.5) for 30 minutes, and incubated with a solution of the radioligand in TBS for 60 min (1 mL per slide). To determine the total binding (TB), the radioligand was diluted with TBS to a concentration of 2 MBq/mL. To determine non-specific binding (NSB), a solution of the nonradioactive reference compound in ethanol (3.5 mM) was added to the radioligand solution and diluted to give a final concentration of 25 μM carrier and an activity concentration of 2 MBq/mL. After incubation, the unbound radioligand was removed by washing the sections in ice-cold TBS (three times for 5 minutes) and water (1 minute). Internal standards were prepared by serial dilution of the radioligand solution. Tissue sections and internal standards absorbed on filter paper were air-dried and subsequently exposed to a phosphor screen (BAS-IP MS; GE Healthcare) overnight.

Phosphorimaging was performed on a Typhoon FLA 7000 Biomolecular Imager (GE Healthcare). All phosphorimaging experiments were performed in at least three adjacent tissue sections per case, surgical specimen, and experimental condition (TB, NSB). Quantification of phosphor images was performed using the analysis software ImageJ.[11] Determination of TB and NSB (in $kBq/cm^2$) was based on correlation curves generated from the internal standards. The specific binding was calculated as the difference between TB and NSB. The contrast of the autoradiograms was adjusted to match the quantitative readout. The scale bar included in figures containing autoradiography images covers the observed range of tracer binding observed in this study (0–180 $kBq/cm^2$). Images are presented following best practice.[12]

### IMMUNOHISTOCHEMICAL STAINING AND HISTOLOGY

To assess aldosterone synthase (CYP11B2) and 11β-hydroxylase (CYP11B1) protein expression, consecutive sections were processed for IHC. Frozen sections were thawed to room temperature and fixed in 10% neutral buffered formalin for 10 minutes, followed by incubation in methanol/hydrogen peroxide (3%) for 10 minutes to block endogenous peroxidase activity. Slides were then incubated in 1% bovine serum albumin for 30 minutes at room temperature to block non-specific binding, and sections were incubated with the primary antibody for 2 hours at room temperature. The primary antibodies used for IHC were mouse-derived anti–aldosterone synthase (Sigma, MABS1251; 1:1000) and rat-derived anti–11β-hydroxylase (Sigma, MABS502; 1:300). After three 5-minute washes in Tris-buffered saline with tween (TBS-T), slides were incubated for 1 hour with the corresponding biotinylated goat-derived IgG secondary antibody (Vector Laboratories BA-9200 and

BA-9400, 1:200). Slides were then washed as before and incubated in pre-conjugated Strept(avidin)–Biotin Complex (ABC; DAKO) for signal amplification. After a final wash, chromogenic detection was performed with 3,3'-diaminobenzidine (DAB), followed by counterstaining with Mayer's hematoxylin. Slides were dehydrated through graded ethanol (70, 90 and 100%), cleared in xylene, and mounted. Sections were scanned using an Olympus VS120 slide scanner at 20x magnification.

To confirm aldosterone synthase positivity in FFPE tissue, sections (8 µm) were cut from each specimen and mounted on SuperFrost Plus slides. Sections were deparaffinized in xylene, rehydrated through graded ethanol, and subjected to heat-induced antigen retrieval by boiling in 0.1 M citrate buffer (pH 6) for 10 minutes. Tissue was then processed as described above. Additional sections were stained with hematoxylin and eosin (H&E) to assess adrenal morphology.

## SUPPLEMENTARY TABLES

Table S1. CORTISOL CO-SECRETION, AVS AND PET RESULTS *

| Participant | ONDST Cortisol (nmol/L) [a)] | AVS LI [c)] | (Dominant) | SUVR | (Dominant) | PET LI (%) | (Dominant) |
|---|---|---|---|---|---|---|---|
| 01 | n.d. | 11.1 | (R) | 7.1 | (R) | 97 | (R) |
| 02 | **65** [b)] | 28.0 | (R) | 12.0 | (R) | 100 | (R) |
| 03 | n.d. | N/A [d)] | (R) | 4.4 | (R) | 100 | (R) |
| 04 | **58** [b)] | 39.6 | (R) | 10.3 | (R) | 100 | (R) |
| 05 | 21 | N/A [b)] | (R) | 10.3 | (R) | 100 | (R) |
| 06 | 34 | 5.8 | (R) | 3.8 | (R) | 99 | (R) |
| 07 | <25 | N/A [d)] | (R) | 1.8 | (R) | 76 | (R) |
| 08 | 43 | 8.8 | (L) | 2.9 | (L) | 97 | (L) |
| 09 | 21 | 115.5 | (R) | 9.5 | (R) | 100 | (R) |
| 10 | 10 | 85.4 | (L) | 13.0 | (L) | 100 | (L) |
| 11 | 24 | 3.9 | (L) | 9.6 | (L) | 100 | (L) |
| 12 | 17 | 4.6 | **(L)** | 1.0 | **(bilateral)** | 69 | **(L)** |
| 13 | 46 | 23.9 | (R) | 6.5 | (R) | 100 | (R) |
| 14 | 25 | 43.6 | (R) | 2.1 | (R) | 100 | (R) |
| 15 | n.d. | 10.1 | (R) | 5.6 | (R) | 100 | (R) |
| 16 | 47 | 361.7 | (L) | 6.4 | (L) | 100 | (L) |
| 17 | 44 | 17.4 | (L) | 11.0 | (L) | 100 | (L) |

* ***Abbreviations***: AVS – adrenal vein sampling, L – left, LI – lateralization index, n.d. – not determined, ONDST – overnight dexamethasone suppression test, PET – positron emission tomography, PET LI – percentage of radioligand uptake (AU = $SUV_{mean}$ x volume) in dominant adrenal = AU (dominant)/(AU (dominant) + AU (contralateral)), R – right, SUVR – ratio of $SUV_{max}$ in dominant over $SUV_{max}$ in contralateral adrenal

***Notes***: [a] Cortisol concentration after overnight dexamethasone suppression test, [b] Cortisol concentration above normal value of 50 nmol/L suggests cortisol co-secretion [c] AVS carried out under ACTH stimulation, [d] Right adrenal vein cannulation was unsuccessful, yet lateralization was supported by a suppressed aldosterone-to-cortisol ratio in the inferior vena cava relative to the left adrenal vein, consistent with right-sided aldosterone excess

Table S2. REPRESENTATIVENESS OF STUDY PARTICIPANTS *

| Sex | | Age | Race | |
|---|---|---|---|---|
| ***Study cohort*** | | | | |
| 7 (41%) **female** | Participants 02, 05, 06, 08, 12, 15, 16 | **52.6** ± 9.6 (range 38–69) | 10 (59%) **White** | Participants 01–03, 05, 06, 08, 09, 13, 15, 17 |
| 10 (59%) **male** | Participants 01, 03, 04, 07, 09–11, 13, 14, 17 | | 4 (24%) **Asian** | Participants 07, 10, 11, 14 |
| | | | 3 (19%) **Black** | Participants 04, 12, 16 |
| ***International cohorts*** | | | | |
| PASO – 311 (44%) female, 394 (56%) male[4] | | PASO – 50.8 ± 10.9[4] | Race distribution of cohorts dependent on study center locations: | |
| PAMO – 610 (48.5%) female, 648 (51.5%) male[5] | | PAMO – 52.0 ± 11.5[5] | PAMO – 445 (35.4%) European, 150 (11.9%) American, 530 (42.1%) Asian, 133 (10.6%) Oceanian[5] | |
| AVIS-2 – 640 (39.4%) female, 985 (60.6%) male[6] | | AVIS-2 – 50.8 ± 10.8[6] | AVIS-2 – 75.2% White, 20.7% Asian, 3.6% African, 0.6% Hispanic[6] | |
| ***PA population*** | | | | |
| PA shows similar prevalence between sexes. Therefore, gender is not considered a relevant modifying factor in this analysis. Although men are slightly overrepresented in large cohort studies, women are more likely to be screened and tend to have better treatment outcomes.[13] | | Most patients are diagnosed when they are between 30 and 60 years of age, in line with the median recruitment age for many studies. | The prevalence of PA appears consistent across ethnic groups.[14] Detection rates vary due to differences in clinical suspicion and and screening practices, with higher screening rates observed in Black populations and lower rates in Asian populations compared with White populations.[13,15] Race-specific polymorphisms affect the sensitivity to aldosterone; affecting clinical outcome but not prevalence.[16] | |
| ***London population*** | | | | |

| | | |
|---|---|---|
| N/A | N/A | 53.8% White, 20.7% Asian, 13.5% Black, 5.7% Mixed, 6.3% Other[7] |

***Overall representativeness of this observational study***

This observational study included participants with PA aged 38 to 69 years (median 52.6), consistent with the typical age range at diagnosis. Female participants comprised 41% of the cohort. The ethnic composition (59% White, 24% Asian, 19% Black) reflects the diversity of the local population and is broadly representative of the global PA population, although Black patients may be underrepresented.

* ***Abbreviations***: AVIS – Adrenal Vein Sampling International Study, PA – primary aldosteronism, PAMO – Primary Aldosteronism Medical Treatment Outcome, PASO – Primary Aldosteronism Surgical Outcome

***Notes***: [x] Literature references in blue – details on page 33

**Table S3. PET-CT FINDINGS ***

| Participant | $SUV_{max}$ (R) | $SUV_{mean}$ (R) | Volume ($cm^3$) (R) | AU (R) | $SUV_{max}$ (L) | $SUV_{mean}$ (L) | Volume ($cm^3$) (L) | AU (L) | Dominant (AU) | PET LI (%) |
|---|---|---|---|---|---|---|---|---|---|---|
| **01** | 34.3 | 11.4 | 4.4 | 50.0 | 4.8 | 4.4 | 0.4 | 1.6 | **R** | **97** |
| | 4.2 | 4.2 | 0.1 | 0.3 | - | - | - | - | - | - |
| **02** | 29.9 | 9.0 | 2.5 | 22.6 | 2.5 | - | - | - | **R** | **100** |
| **03** | 8.3 | 5.2 | 6.8 | 35.8 | 1.9 | - | - | - | **R** | **100** |
| **04** | 30.0 | 9.5 | 3.3 | 31.4 | 3.7 | - | - | - | **R** | **100** |
| | 5.5 | 4.5 | 0.3 | 1.2 | - | - | - | - | - | - |
| | 25.6 | 7.8 | 2.6 | 20.1 | - | - | - | - | - | - |
| **05** | 32.6 | 9.8 | 5.7 | 55.4 | 3.2 | - | - | - | **R** | **100** |
| **06** | 25.0 | 9.7 | 8.6 | 83.4 | 6.6 | 5.0 | 0.2 | 1.0 | **R** | **99** |
| **07** | 15.5 | 6.6 | 1.8 | 11.6 | 8.8 | 5.2 | 0.7 | 3.7 | **R** | **76** |
| **08** | 5.9 | 4.8 | 0.6 | 2.8 | 17.2 | 8.5 | 9.3 | 79.7 | **L** | **97** |
| **09** | 25.8 | 8.2 | 2.3 | 18.9 | 2.7 | - | - | - | **R** | **100** |
| **10** | 2.0 | - | - | - | 26.3 | 9.0 | 1.8 | 16.2 | **L** | **100** |
| **11** | 2.6 | - | - | - | 25.4 | 8.9 | 2.0 | 18.1 | **L** | **100** |
| **12** | 6.7 | 5.0 | 0.8 | 4.1 | 6.7 | 5.1 | 0.3 | 1.5 | **L** | **69** |
| | - | - | - | - | 5.9 | 4.6 | 1.7 | 7.8 | - | - |
| **13** | 22.1 | 9.0 | 7.0 | 62.8 | 3.4 | - | - | - | **R** | **100** |
| **14** | 7.0 | 5.1 | 0.6 | 3.0 | 3.4 | - | - | - | **R** | **100** |
| **15** | 18.9 | 7.5 | 1.9 | 14.5 | 3.4 | - | - | - | **R** | **100** |
| **16** | 3.8 | - | - | - | 24.6 | 9.5 | 8.5 | 80.5 | **L** | **100** |

| | | | | | | | | | | |
|---|---|---|---|---|---|---|---|---|---|---|
| **17** | 2.8 | - | - | - | 30.5 | 9.5 | 3.2 | 30.8 | **L** | **100** |
| | - | - | - | - | 8.2 | 5.3 | 0.8 | 4.0 | - | - |

***** ***Abbreviations***: AU – radioligand uptake = $SUV_{mean}$ x volume, L – left, LI – lateralization index, PET – positron emission tomography, PET LI – percentage of AU in dominant adrenal = AU (dominant)/(AU (dominant) + AU (contralateral)), R – right, SUV – standardized uptake value, SUVR – ratio of $SUV_{max}$ in dominant over $SUV_{max}$ in contralateral adrenal

Table S4. BIOCHEMICAL AND CLINICAL RESPONSE TO ADRENALECTOMY *

| Participant | PET LI (%) | AVS LI [a)] | Biochemical 6m [c)] | Clinical 6m [c)] | Biochemical 12m [c)] | Clinical 12m [c)] |
|---|---|---|---|---|---|---|
| *Participants with asymmetric disease by PET* | | | | | | |
| 01 | 97 | 11.1 | Absent | Partial | *n.a.* | Partial |
| 06 | 99 | 5.8 | Complete | Complete | Complete | Complete |
| 07 | 76 | N/A [b)] | Complete [d)] | Partial | *n.a* | Partial |
| 08 | 97 | 8.8 | Complete | Partial | Complete | Complete |
| 12 | 69 | 4.6 | Partial | Partial | Partial | Partial |
| *Participants with unilateral disease by PET* | | | | | | |
| 02 | 100 | 28.0 | Complete | Complete | Complete | Complete |
| 03 | 100 | N/A [b)] | *n.a.* | *n.a.* | Complete | Partial |
| 04 | 100 | 39.6 | Partial | Partial | Partial | Partial |
| 05 | 100 | N/A [b)] | Complete | Complete | Complete | Complete |
| 09 | 100 | 115.5 | Complete | Complete | Complete | Complete |
| 10 | 100 | 85.4 | Complete | Partial | Complete | Partial |
| 11 | 100 | 3.9 | Complete | Partial | Complete | Partial |
| 13 | 100 | 23.9 | Complete | Partial | Complete | Partial |
| 14 | 100 | 43.6 | Complete | Partial | Complete | Partial |
| 15 | 100 | 10.1 | Partial | Partial | *n.a.* | Partial |
| 16 | 100 | 361.7 | Complete | Complete | *n.a.* | Complete |
| 17 | 100 | 17.4 | Complete | Partial | Complete | Partial |

***** ***Abbreviations***: AVS – adrenal vein sampling, LI – lateralization index, n.a. – not assessed, PET – positron emission tomography, PET LI – percentage of radiotracer uptake (AU = $SUV_{mean}$ x volume) in dominant adrenal = AU (dominant)/(AU (dominant) + AU (contralateral))

***Notes***: [a)] AVS carried out under ACTH stimulation, [b)] Right adrenal vein cannulation was unsuccessful, yet lateralization was supported by a suppressed aldosterone-to-cortisol ratio in the inferior vena cava relative to the left adrenal vein, consistent with right-sided aldosterone excess, [c)] Biochemical and clinical response as per PASO criteria[4], [d)] Assessed 4 months after surgery

## SUPPLEMENTARY FIGURES

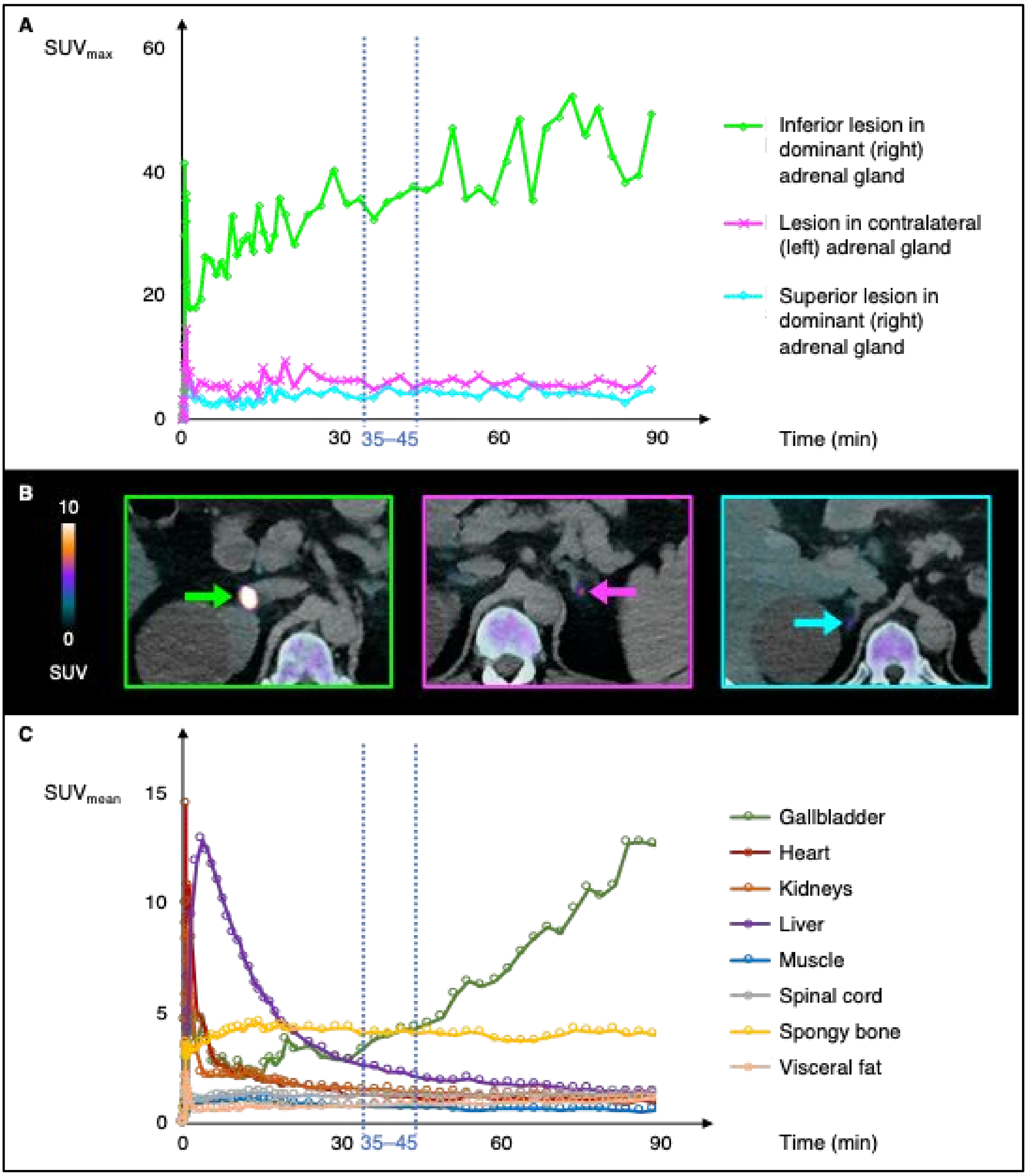


**Figure S1. Distribution of radioligand uptake over time**

Time-activity curves of radioligand uptake in the adrenal lesions (**panel A**) and in organs adjacent to the adrenal glands (**panel C**) of participant 01 show high signal in the dominant adrenal lesion and comparable, lower uptake in bilateral microlesions. The imaging window (blue dashed lines) for static scans was selected based on the uptake

profile in adrenal lesions and radiotracer clearance from surrounding organs. Time-activity curves were created from dynamic PET data acquired over 90 minutes immediately post administration using PMOD (Version 3.5, Zurich, Switzerland). PET-CT images covering the three lesions are shown in **panel B** for comparison.

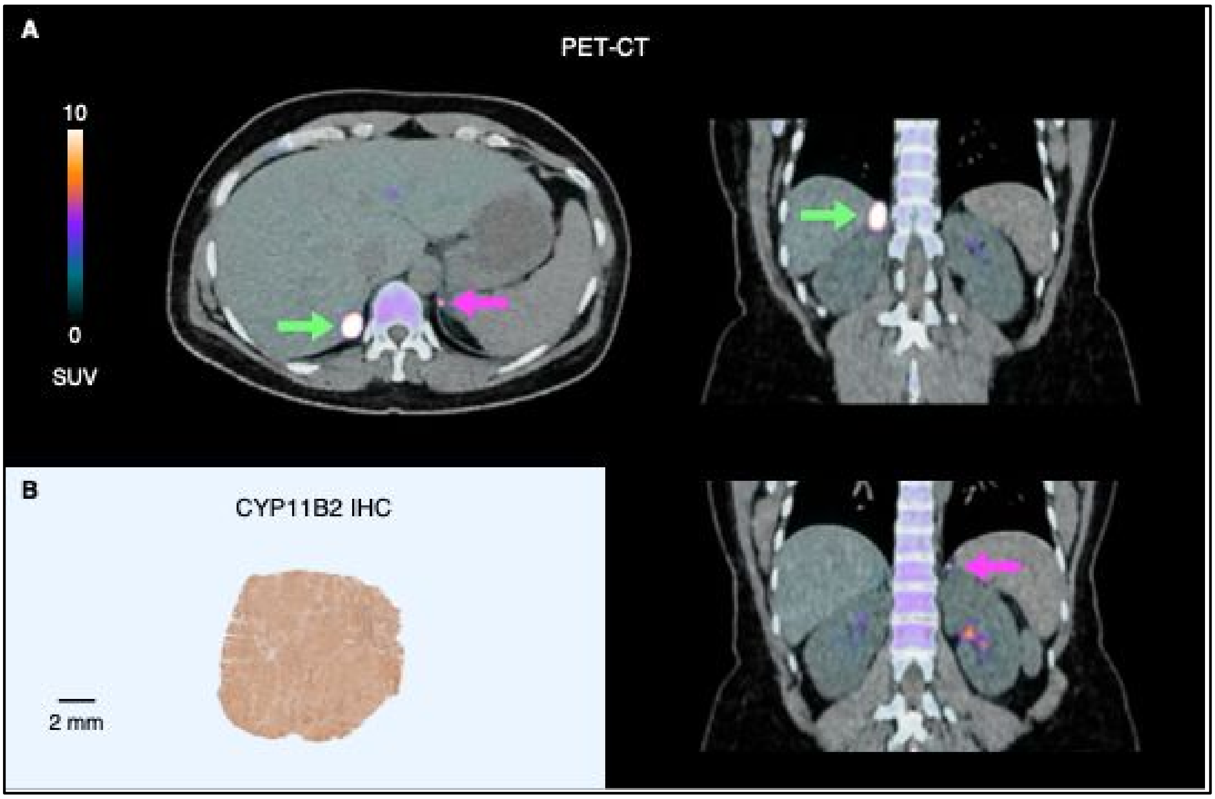


**Figure S2. Participant 06**

The participant (female, White, 43 years of age) had a dominant lesion (**panel A**, green arrows) in the right adrenal and a microlesion (pink arrows) in the contralateral gland. The SUVR was 3.8 and the PET LI was 99%. AVS lateralized to the right (AVS LI = 5.8). The participant underwent adrenalectomy of the right adrenal gland. Excretion of the radioligand in the renal pelvis can be seen.

Aldosterone synthase (CYP11B2) specific IHC staining (**panel B**) confirmed abnormal enzyme expression in the specimen taken from the surgically removed, right adrenal (frozen tissue).

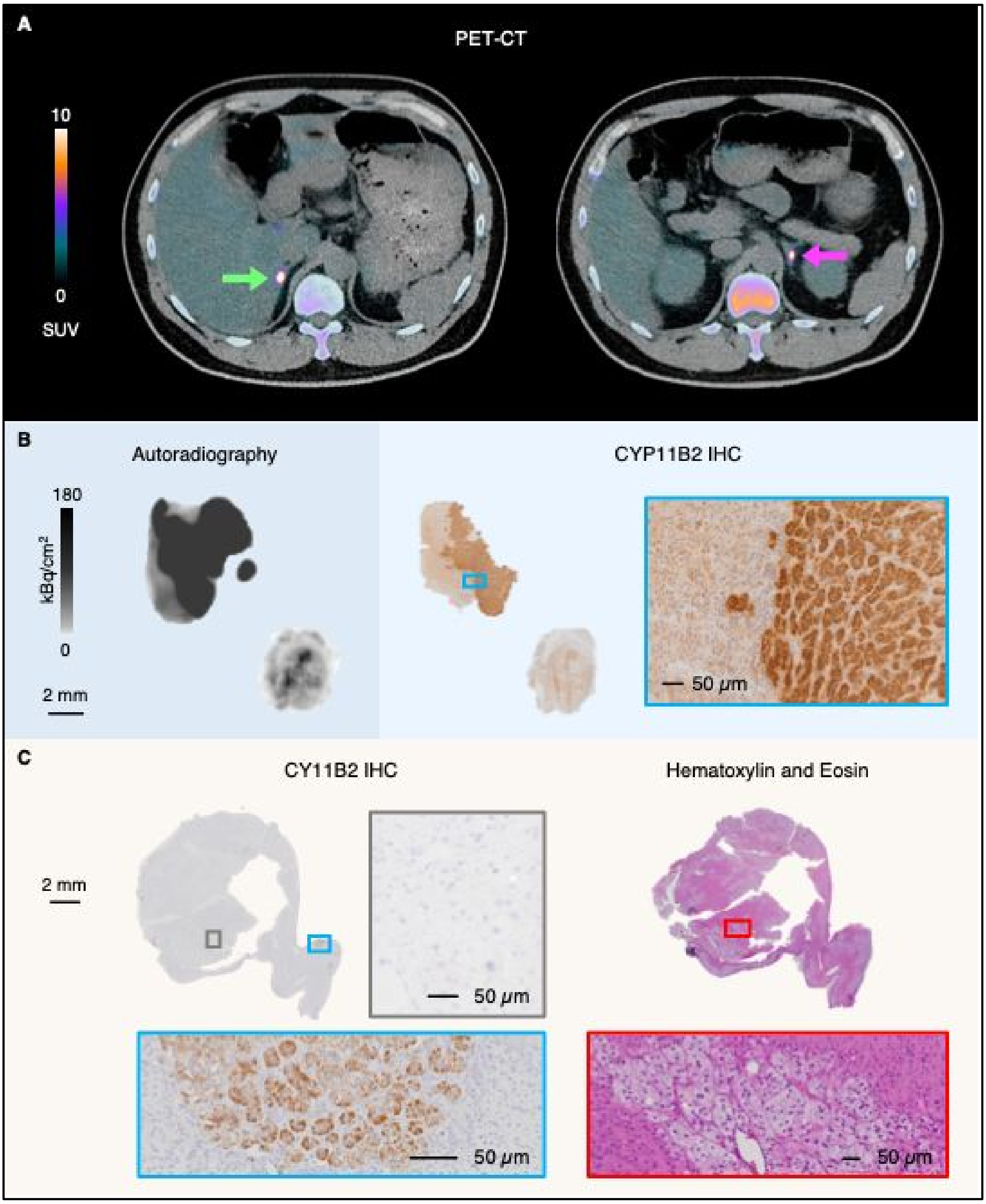


**Figure S3. Participant 07**

The participant (male, Asian, 41 years of age) had a dominant lesion in the right adrenal (**panel A**, green arrow) and a microlesion in the contralateral gland (pink arrow). The SUVR was 1.8 and the PET LI was 76%. AVS cannulation of the right vein was unsuccessful, but the left adrenal was suppressed. The participant had adrenalectomy of the right adrenal gland. Autoradiography in freshly frozen tissue sections was consistent with the

binding pattern determined by IHC staining of aldosterone synthase in adjacent sections (**panel B**). The specimen shown in the upper row was taken from the site of the suspected APA, whereas the other specimen was taken from a distant site.

**Figure S3. Participant 07,** ***continued***

Serially sectioned fixed tissue (**panel C**) revealed an additional lesion with disorganized cell structure and arrangement in the hematoxylin and eosin stain that was negative for aldosterone synthase (grey magnification), apart from a micronodule in the adjacent adrenal cortex (cyan magnification). The findings are consistent with the results from *in vivo* imaging, which showed that the PET positive lesion in the dominant adrenal did not co-localize with the nodule detected by CT (**Figure S7** and **Figure S8**).

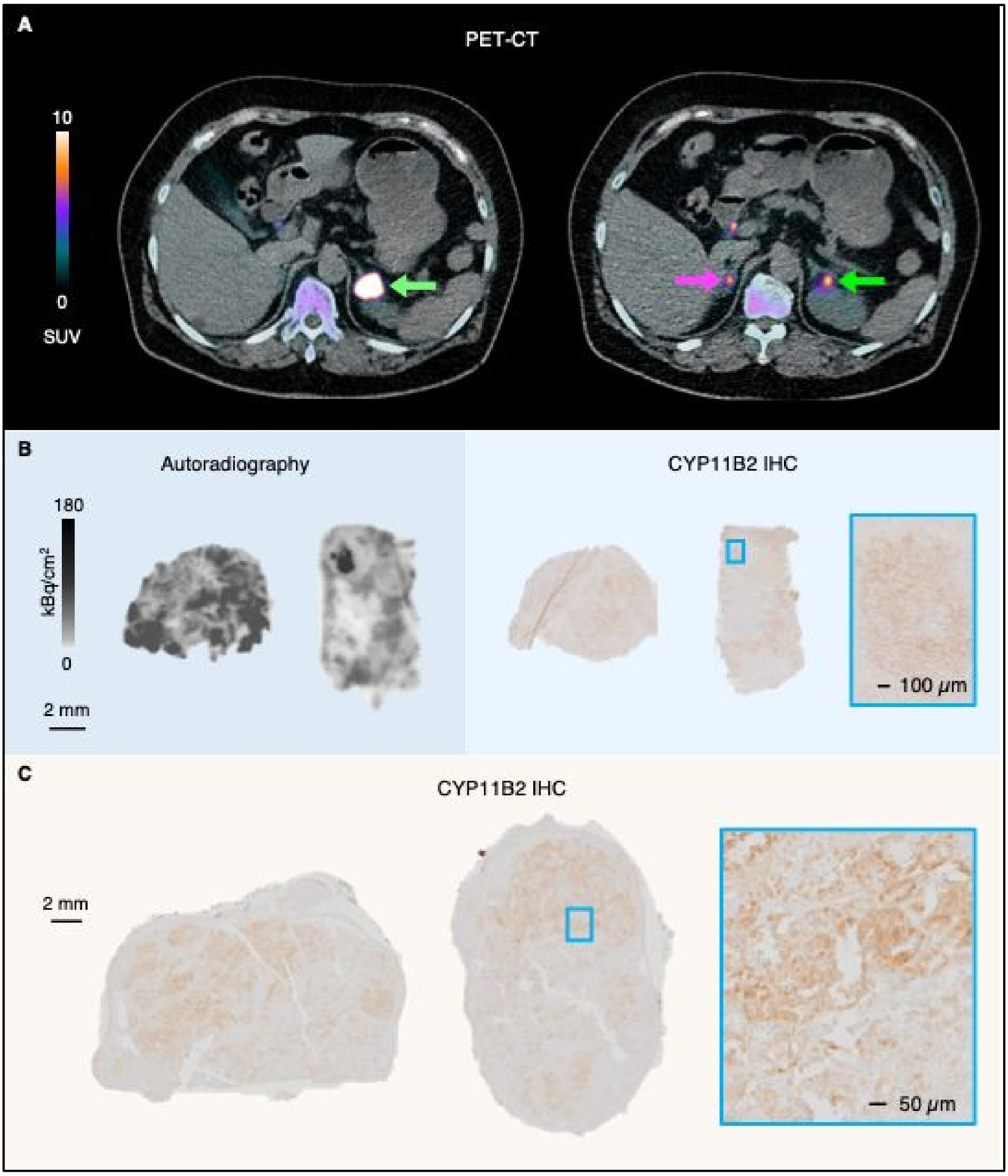


**Figure S4. Participant 08**

The participant (female White, 66 years of age) had a dominant lesion in the left adrenal (**panel A**, green arrows) and a microlesion in the right adrenal (pink arrow). The SUVR was 2.9 and PET LI was 97%. AVS lateralized to the left (AVS LI = 8.8). The participant had adrenalectomy of the left adrenal gland. Excretion of the radiotracer in the hepatic duct can be seen.

Autoradiography in sections from freshly frozen tissue (**panel B**) showed patterns of moderate, heterogenous tracer binding in sections sampled from two distinct sites, including the suspected APA. Given the large lesion identified by PET-CT imaging, the surgically resected gland was further assessed using aldosterone synthase (CYP11B2) specific IHC staining in serially sectioned fixed tissue (**panel C**). Moderate staining was observed in multiple tissue blocks (two examples shown) consistent with an APA.

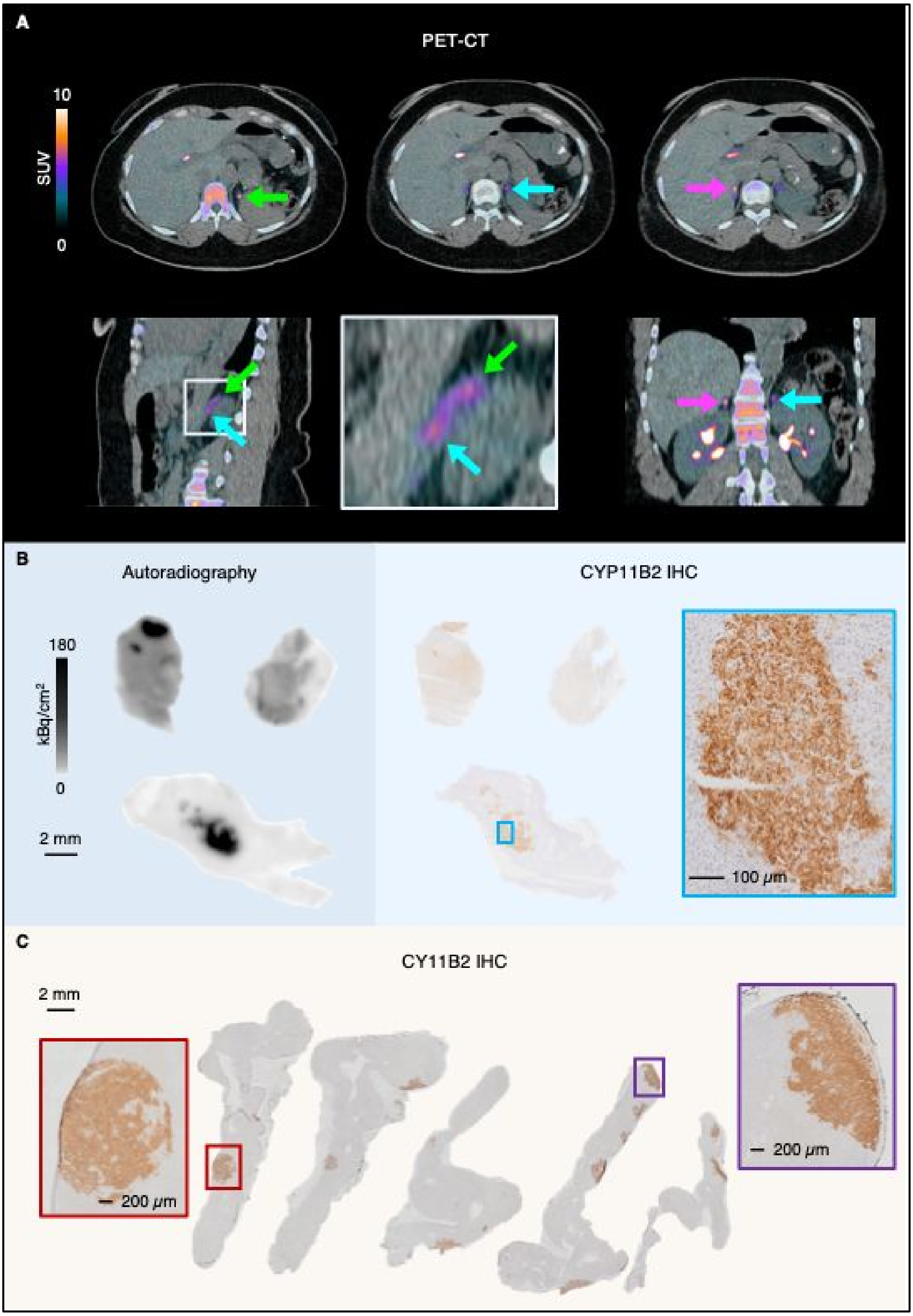
A
PET-CT
10
SUV
0
B
Autoradiography
180
kBq/cm²
0
2 mm
CYP11B2 IHC
100 μm
C
CY11B2 IHC
2 mm
200 μm
200 μm

**Figure S5. Participant 12**

**Figure S5. Participant 12,** ***continued***

The participant (female, Black, 42 years of age) had a dominant lesion (**panel A**, green arrows and magnification) in the left adrenal and bilateral microlesions (cyan, left adrenal and pink, right adrenal). The SUVR was 1.0 and the PET LI was 69%. AVS lateralized to the left (AVS LI = 4.6). The participant had adrenalectomy of the left adrenal gland. Excretion of the radioligand in the hepatic duct and kidney bed can be seen.

Autoradiography in sections from freshly frozen tissue (**panel B**) showed high focal radioligand binding in small areas and low binding to the surrounding tissue. Aldosterone synthase (CYP11B2) specific IHC in adjacent tissue sections confirmed highly localized aldosterone synthase expression consistent with aldosterone-producing micronodules. IHC staining of serially sectioned fixed tissue (**panel C**) revealed additional micronodules with a diameter ranging between 200 μm to 2 mm.

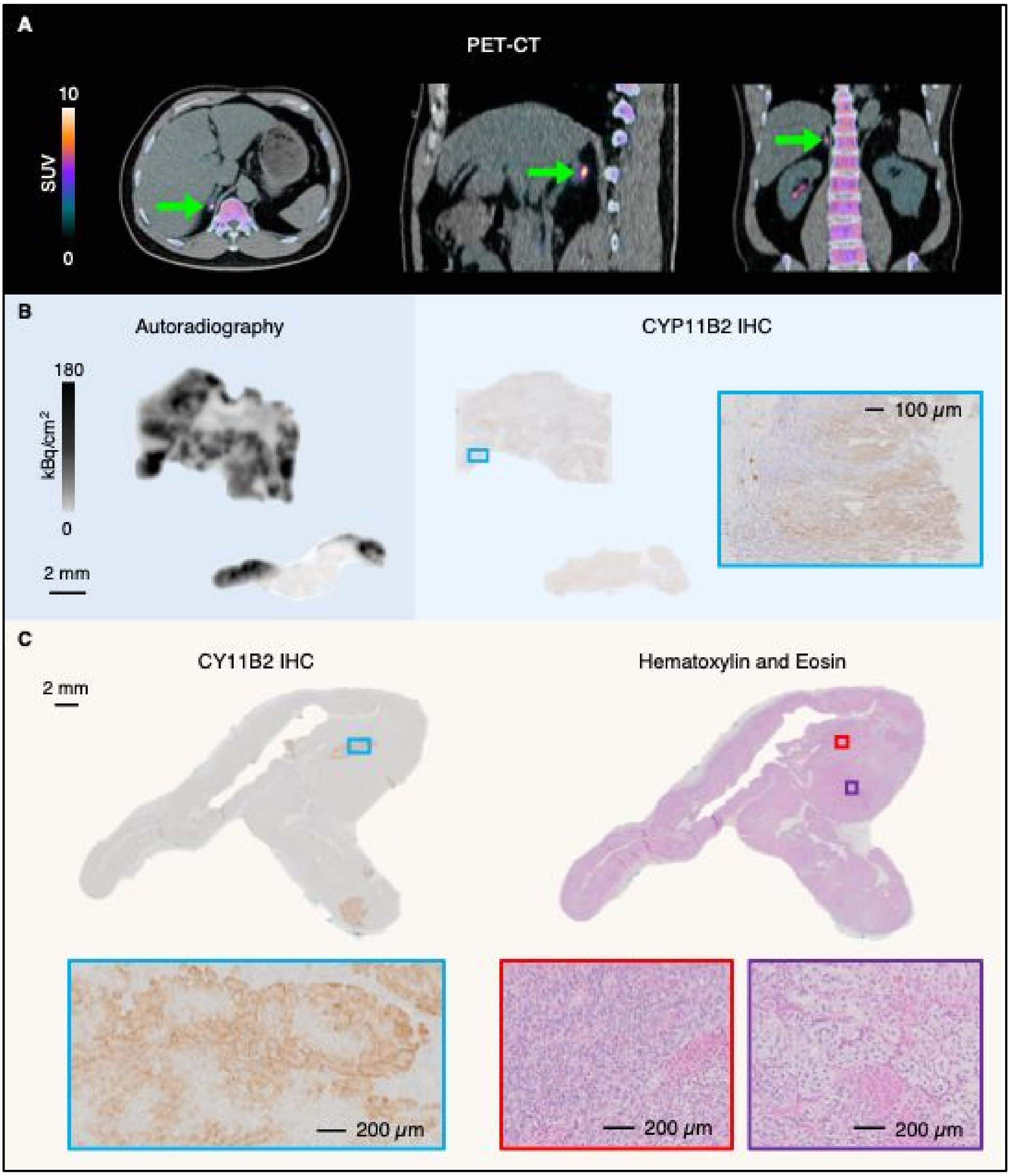


**Figure S6. Participant 14**

The participant (male, Asian, 56 years of age) had a single microlesion in the right adrenal (**panel A**, green arrows). The SUVR was 2.1 and the PET LI was 100%. AVS lateralized to the right (AVS LI = 43.6). Following adrenalectomy of the right adrenal gland, autoradiography in sections from freshly frozen tissue showed heterogenous tracer distribution, consistent with the focal aldosterone synthase (CYP11B2) expression observed with IHC in both frozen (**panel B**) and fixed (**panel C**) tissue sections.

The PET lesion was negative by CT imaging (**Figure S7**), but two nodules were detected by CT at a different location in the dominant adrenal (**Figure S8**). Consistent with this, an aldosterone synthase (CYP11B2) negative lesion with abnormal cellular structure and organization, and hyperplasia in the zona glomerulosa, was identified in formalin-fixed paraffin embedded tissue (**panel C**).

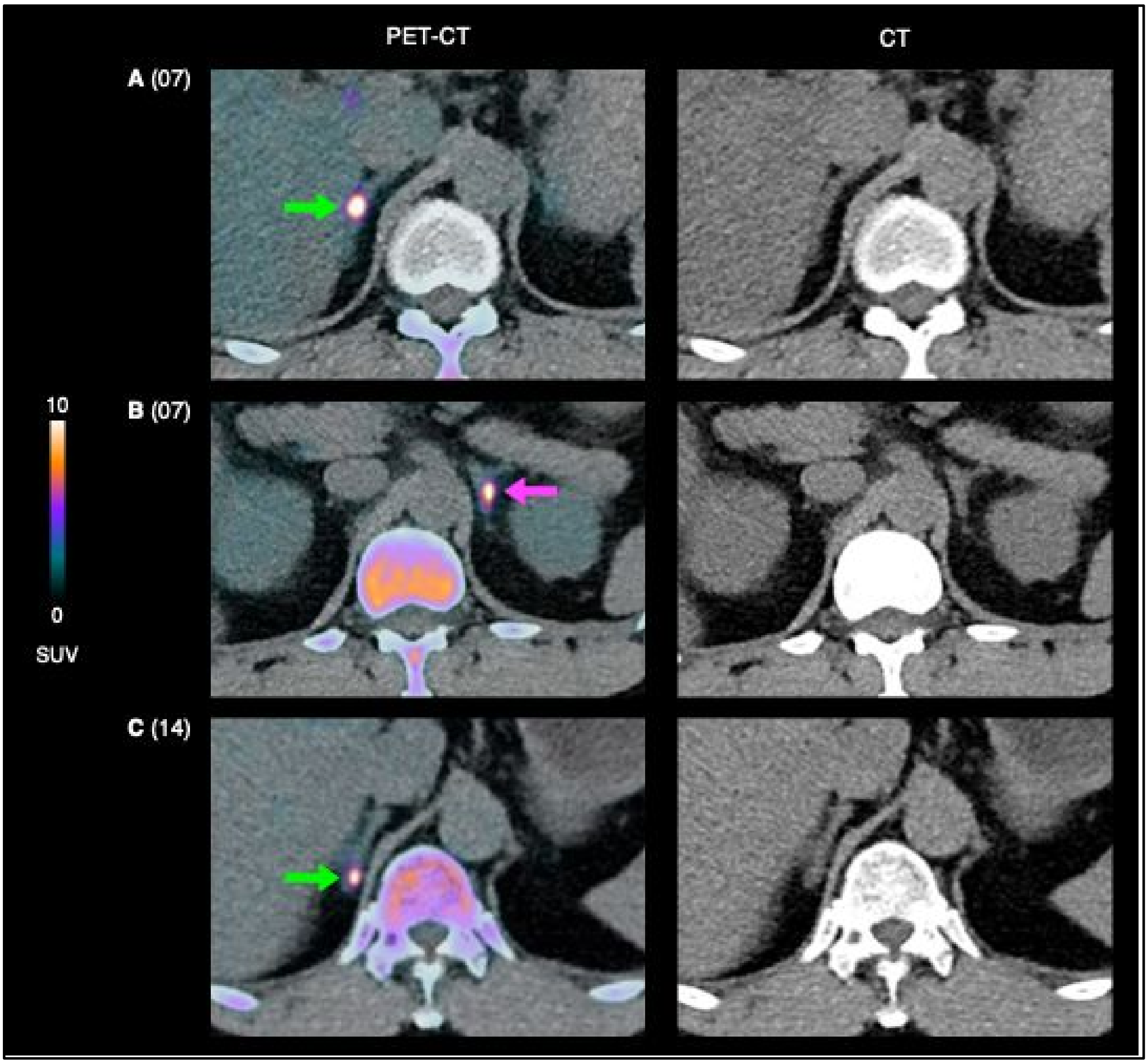


**Figure S7. Adrenal lesions detected by PET that were not seen on CT**

**Panels A** and **B** show a lesion in the adrenal gland predicted to be dominant by AVS and a microlesion in the contralateral adrenal gland, respectively, for the same participant (07). **Panel C** shows a microlesion in the right adrenal gland (participant 14). In the cases displayed, the lesions detected by PET-CT were not identified by diagnostic CT imaging. For both participants, the adrenal nodules observed on CT were negative on PET-CT (**Figure S8**).

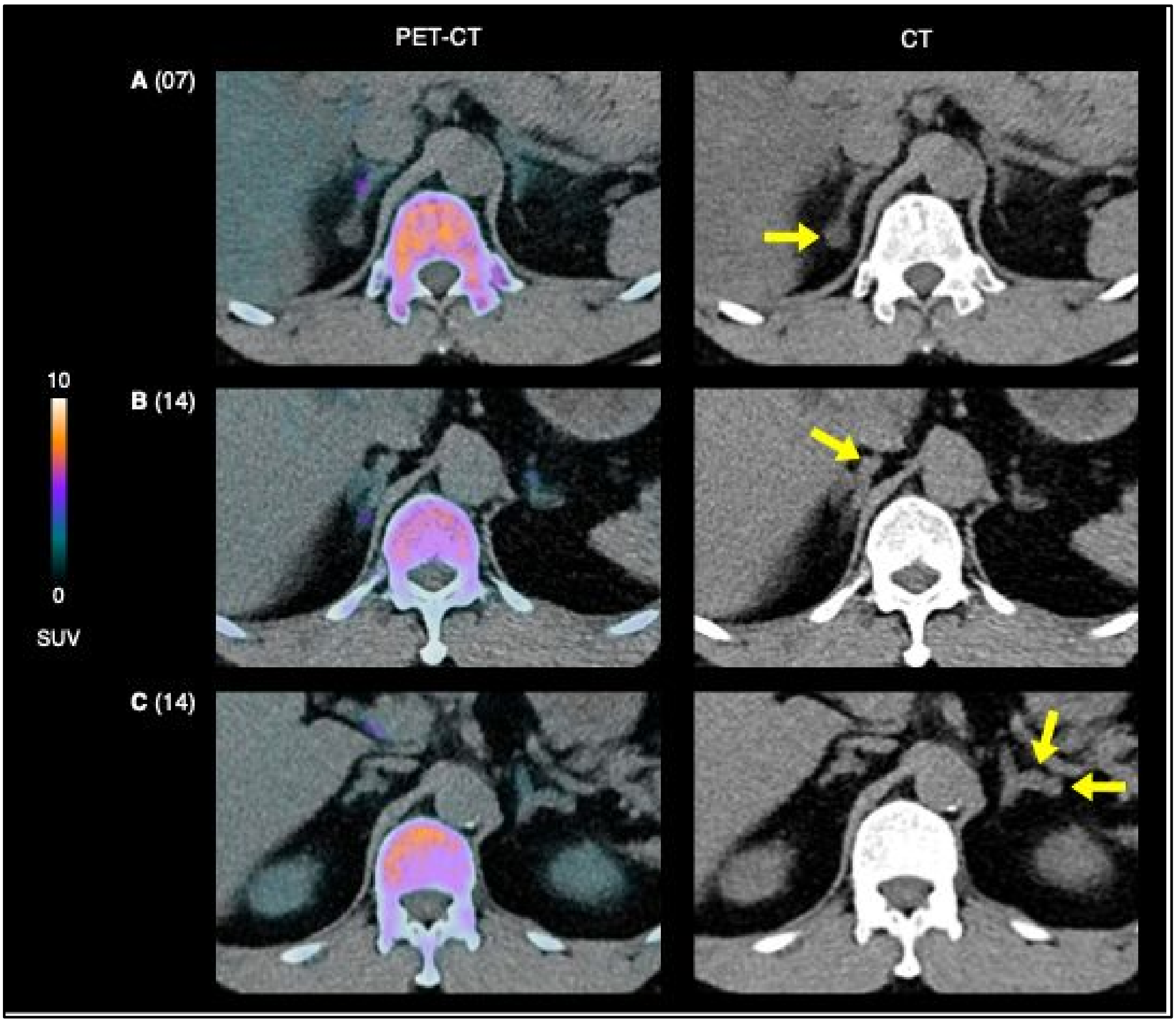


**Figure S8. Absence of radiotracer uptake in CT positive nodules**

Nodules detected by CT imaging (yellow arrows) that were negative by PET-CT were observed in the right (dominant) adrenal gland of participant 07 (**panel A**), and both the dominant (**panel B**) and contralateral (**panel C**) adrenal glands of participant 14.

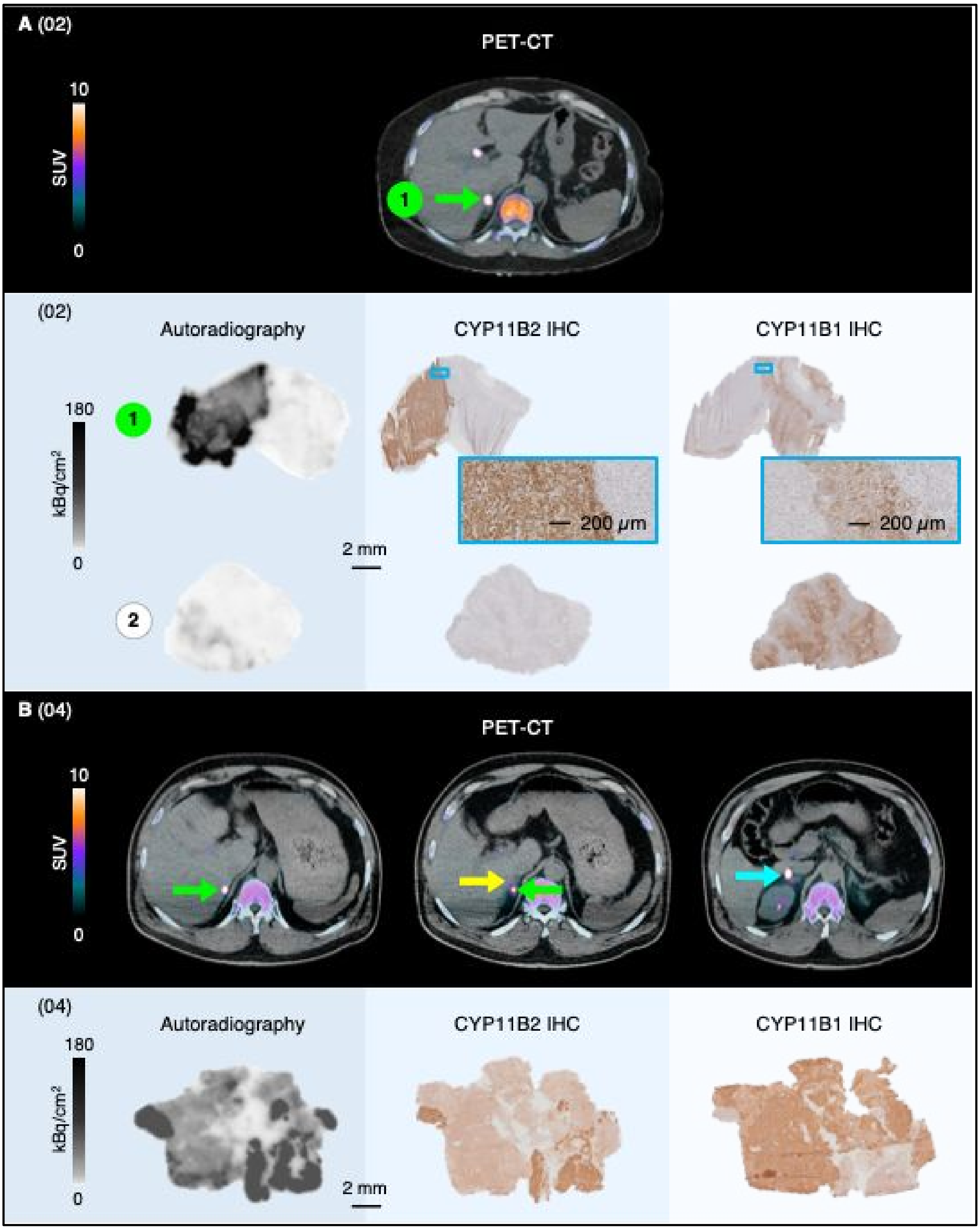


**Figure S9. Radioligand binding in adrenal tissue from participants with cortisol co-secretion**

**Panel A** shows data from participant 02: a single adrenal lesion was detected with PET-CT (***upper panel***, green arrow); tracer binding in specimen 1 and 2 of the surgically resected adrenal gland (*left*), aldosterone synthase (CYP11B2) specific IHC staining (*middle*) and11β-hydroxylase (CYP11B1) staining (*right*) in the directly adjacent

part of the tissue. **Panel B** shows data from participant 04: three distinct adrenal lesions were detected with PET-CT; clusters of tracer binding in the surgically resected gland (*left*) were consistent with CYP11B2 expression (*middle*), but distinct from CYP11B1 (*right*).

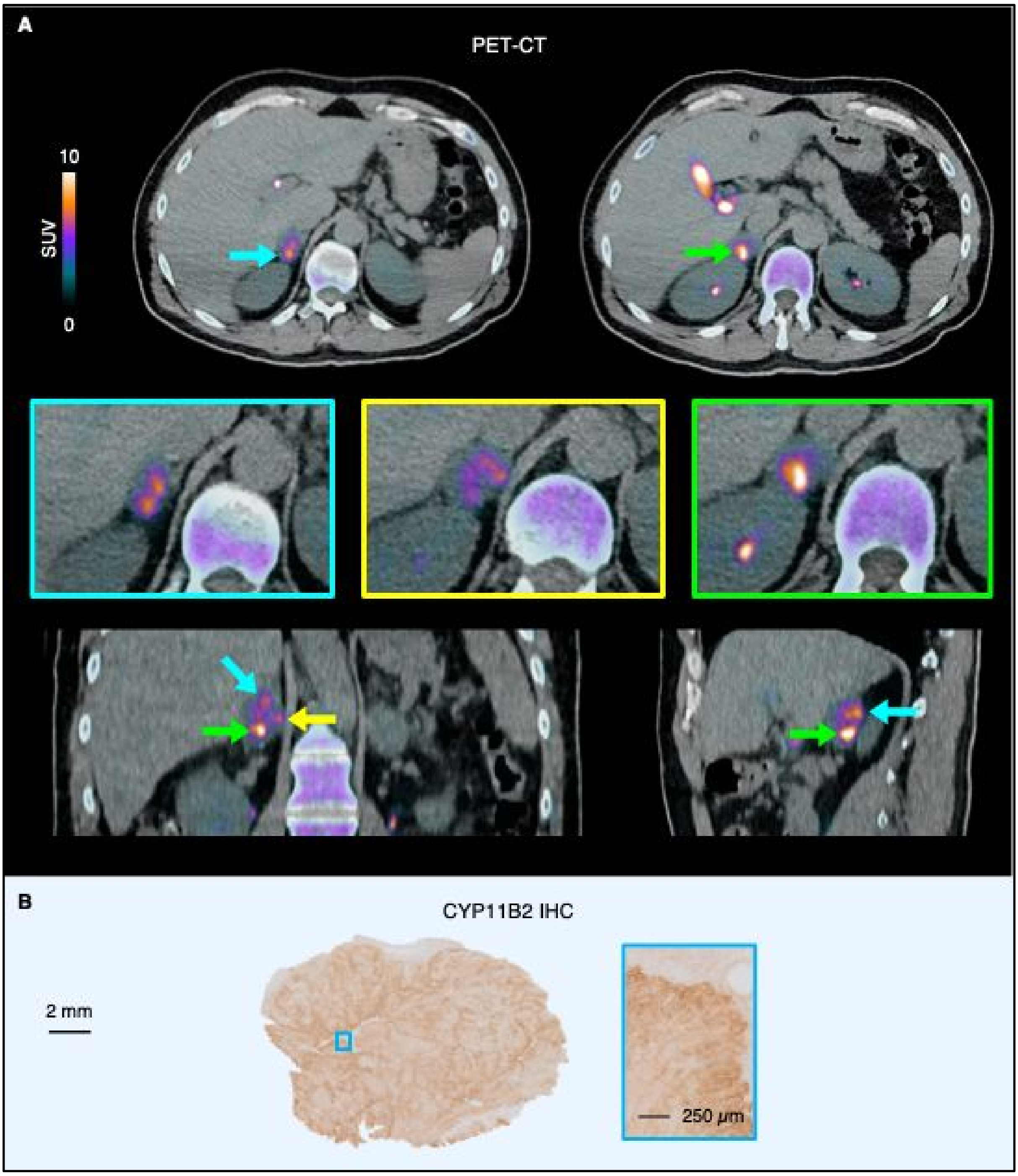


**Figure S10. Participant 03**

The participant (male, White, 52 years of age) had a heterogenous lesion in the right adrenal gland (**panel A**). The SUVR (right/left) was 4.4 and the PET LI was 100%. AVS cannulation of the right vein was unsuccessful, but the left adrenal was suppressed. The participant had adrenalectomy of the right adrenal gland. Excretion of the radiotracer in the hepatic duct and renal pelvis can be seen in images in the axial plane (upper row).

Aldosterone synthase (CYP11B2) specific IHC staining in frozen tissue from the surgically removed gland (**panel B**) confirmed abnormal enzyme expression.

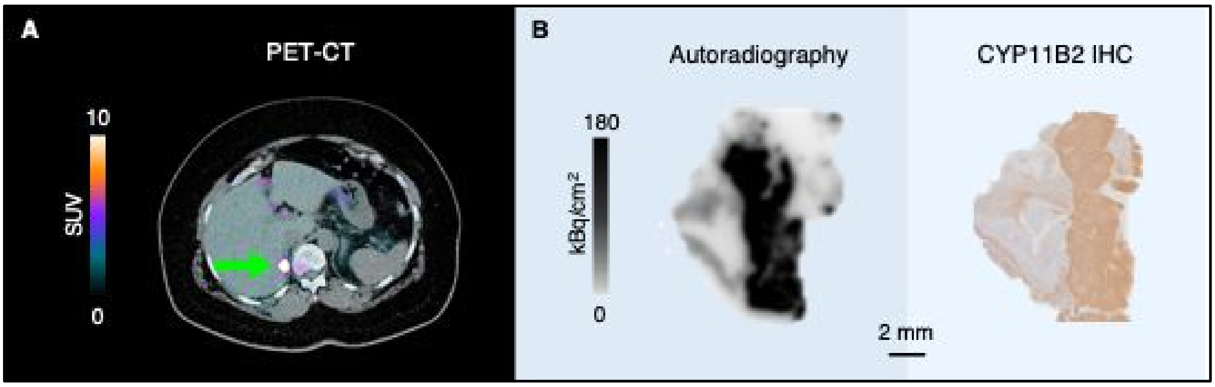


**Figure S11. Participant 05**

The participant (female, White, 54 years of age) had a single lesion in the right adrenal gland (**panel A**, green arrow). The SUVR (right/left) was 10.3 and the PET LI was 100%. AVS cannulation of the right vein was unsuccessful, but the left adrenal was suppressed. The participant had adrenalectomy of the right adrenal gland. In autoradiography experiments, dense radioligand binding was observed in frozen tissue samples from the resected adrenal gland (**panel B**). Radioligand binding co-localized with IHC staining of aldosterone synthase (CYP11B2).

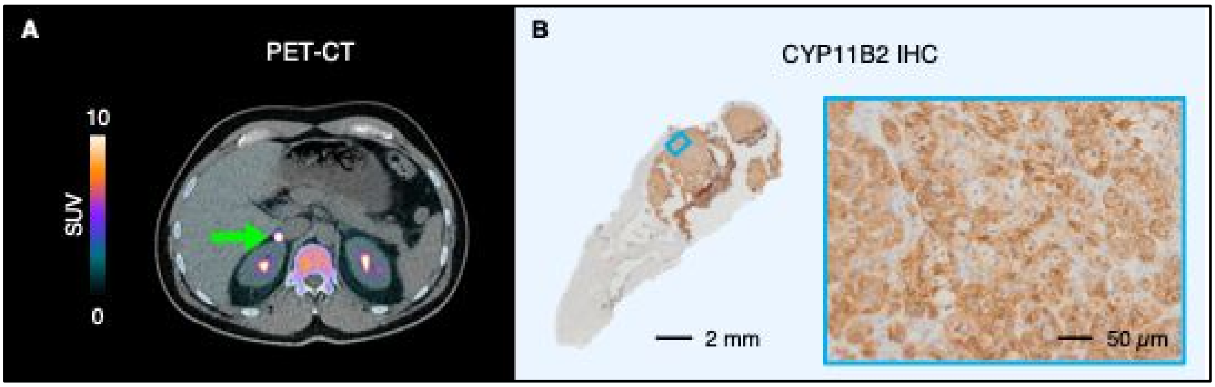


**Figure S12. Participant 09**

The participant (male, White, 50 years of age) had a single lesion in the right adrenal (**panel A**, green arrow). The SUVR was 9.5 and the PET LI was 100%. AVS lateralized to the right (AVS LI = 115.5). The participant had adrenalectomy of the right adrenal gland. Excretion of the radioligand in the kidney bed can be seen. IHC in fixed tissue from the surgically resected adrenal gland (**panel B**) showed increased aldosterone synthase (CYP11B2) expression levels consistent with an APA.

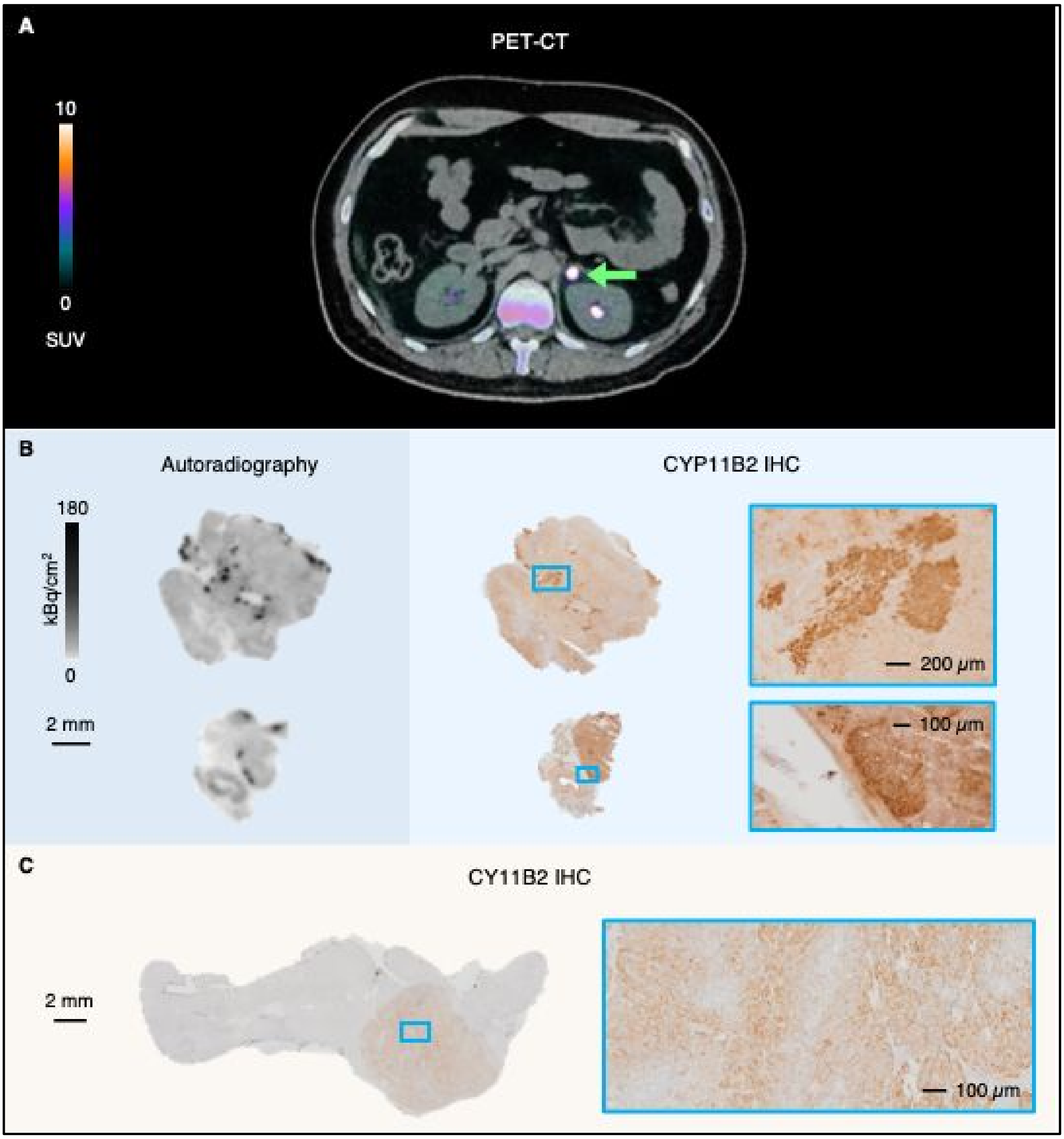


**Figure S13. Participant 10**

The participant (male, Asian, 41 years of age) had a single lesion in the left adrenal (**panel A**, green arrow). The SUVR was 13.0 and the PET LI was 100%. AVS lateralized to the left (AVS LI = 85.4). The participant had adrenalectomy of the left adrenal gland. Excretion of the radiotracer in the kidney bed can be seen.

Autoradiography in sections from freshly frozen tissue (**panel B**) showed patterns of heterogenous radioligand binding, consistent with the expression of aldosterone synthase (CYP11B2). However, the overall tracer binding appeared low compared to the radioligand uptake observed *in vivo* by PET-CT. Staining of serially sectioned fixed tissue subsequently confirmed an aldosterone synthase (CYP11B2) positive lesion consistent with an APA (**panel C**).

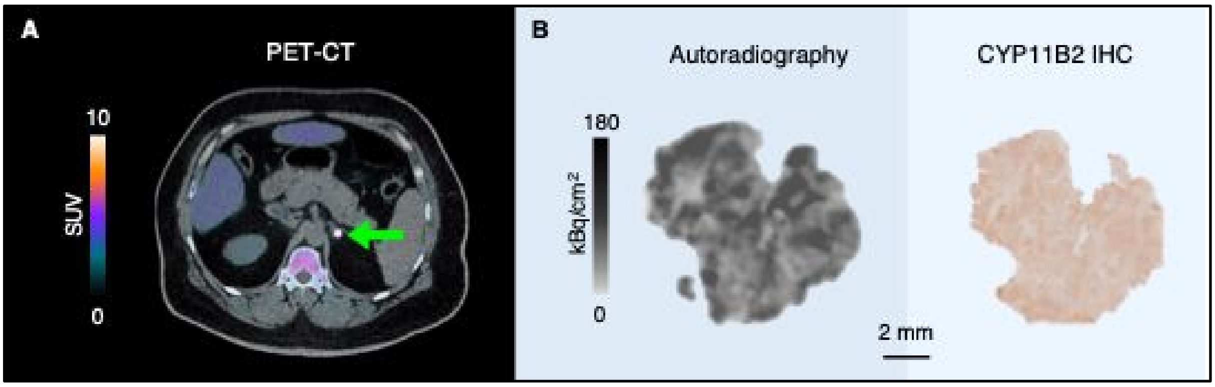


**Figure S14. Participant 11**

The participant (male, Asian, 53 years of age) had a single lesion in the left adrenal gland (**panel A**, green arrow). The SUVR was 9.6 and the PET LI was 100%. AVS was borderline with lateralization to the left (AVS LI = 3.9 vs. cut-off 4.0). The participant had adrenalectomy of the left adrenal gland. Autoradiography in sections from freshly frozen tissue (**panel B**) showed patterns of heterogenous radioligand binding, consistent with the expression of aldosterone synthase (CYP11B2).

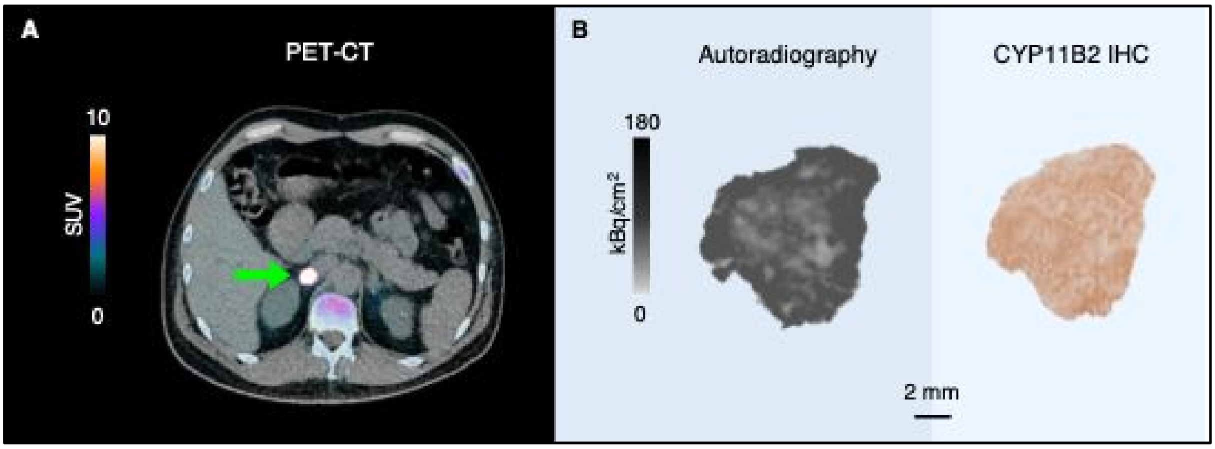


**Figure S15. Participant 13**

The participant (male, White, 43 years of age) had a single lesion (**panel A**, green arrows) in the right adrenal. The SUVR was 6.5 and the PET LI was 100%. AVS lateralized to the right (AVS LI = 23.9). The participant had adrenalectomy of the right adrenal gland. Autoradiography in sections from freshly frozen tissue (**panel B**) showed patterns of heterogenous radioligand binding, consistent with the expression of aldosterone synthase (CYP11B2).

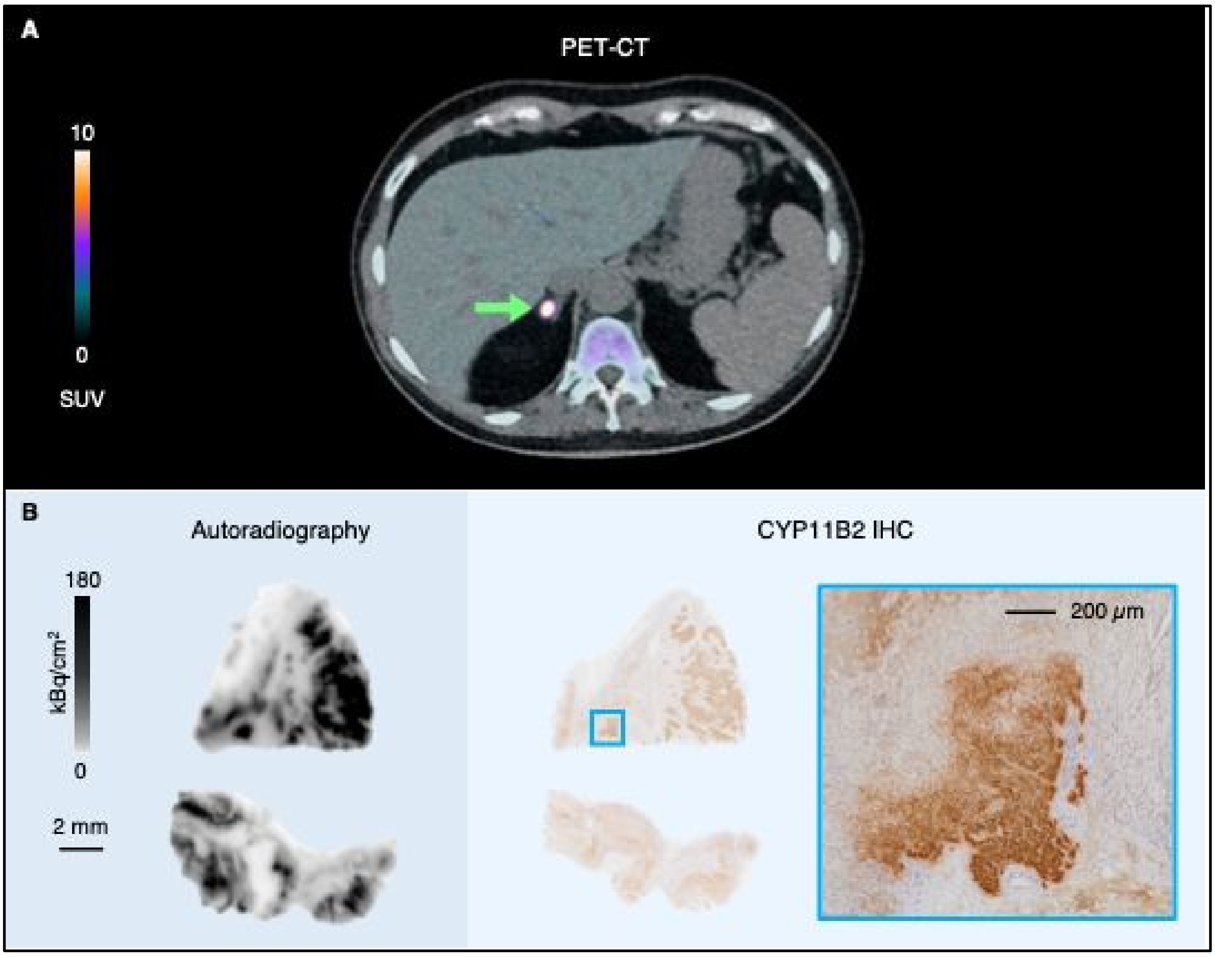


**Figure S16. Participant 15**

The participant (female, White, 62 years of age) had a single lesion in the right adrenal (**panel A**, green arrow). The SUVR was 5.6 and the PET LI was 100%. AVS lateralized to the right (AVS LI = 10.1). The participant had adrenalectomy of the right adrenal gland. Autoradiography in sections from freshly frozen tissue showed heterogenous tracer distribution (**panel B**). The binding pattern is consistent with the expression of aldosterone synthase as determined by IHC in adjacent tissue sections.

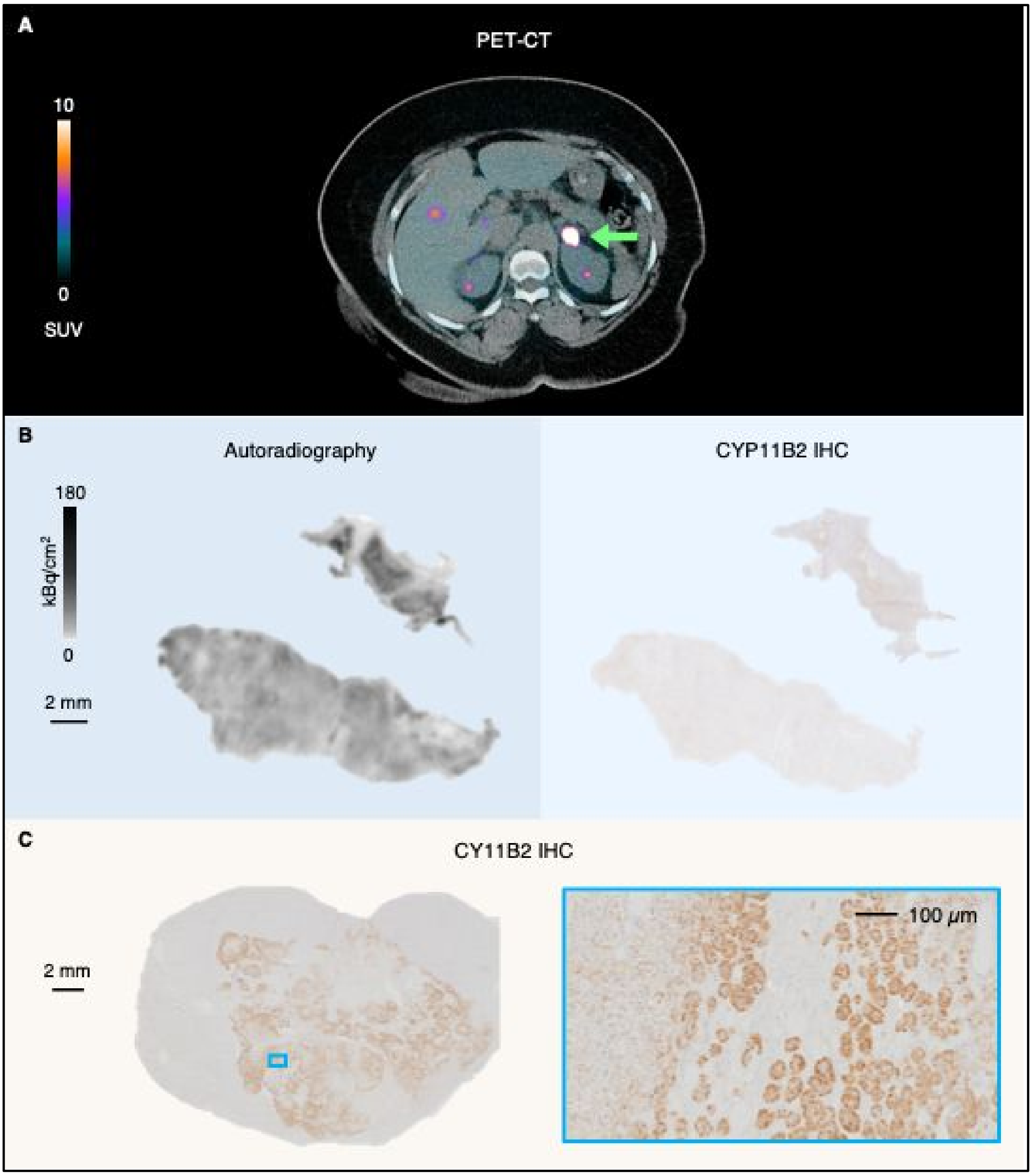


**Figure S17. Participant 16**

The participant (female, Black, 60 years of age) had a single lesion in the left adrenal (**panel A**, green arrow). The SUVR was 6.4 and the PET LI was 100%. AVS lateralized to the left (AVS LI = 361.7). The participant had adrenalectomy of the left adrenal gland. Excretion of the radiotracer in the hepatic duct and the kidney bed can be seen.

Frozen tissue sampled from the site of the suspected APA showed low radioligand binding and low aldosterone synthase expression as determined by IHC in adjacent tissue sections (**panel B**). IHC staining of serially sectioned fixed tissue (**panel C**) subsequently confirmed a large aldosterone synthase positive lesion consistent with an APA.

## REFERENCES


1. Rossi GP, Auchus RJ, Brown M *et al*. An expert consensus statement on use of adrenal vein sampling for the subtyping of primary aldosteronism. *Hypertension*. **2014**, *63(1)*: 151–160.
2. Sander K, Gendron T, Cybulska KA *et al*. Development of [$^{18}$F]AldoView as the first highly selective aldosterone synthase PET tracer for imaging of primary hyperaldosteronism. *J Med Chem*. **2021**, *64(13)*: 9321–9329.
3. Hoyt SB, Park MK, London C *et al*. Discovery of benzimidazole CYP11B2 inhibitors with in vivo activity in Rhesus monkeys. *ACS Med Chem Lett*. **2015**, *6(5)*: 573–578.
4. Williams TA, Lenders JWM, Mulatero P *et al.* Outcomes after adrenalectomy for unilateral primary aldosteronism: an international consensus on outcome measures and analysis of remission rates in an international cohort. *Lancet Diabetes Endocrinol.* **2017**, *5*: 689–699.
5. Yang J, Burrello J, Goi J *et al.* Outcomes after medical treatment for primary aldosteronism: an international consensus and analysis of treatment response in an international cohort. *Lancet Diabetes Endocrinol.* **2025**, *13(2)*: 119–133.
6. Rossitto G, Amar L, Azizi M, *et al*. Subtyping of primary aldosteronism in the AVIS-2 study: assessment of selectivity and lateralization. *J Clin Endocrinol Metab*. **2020**, *105(6)*: dgz017.
7. UK Office for National Statistics. UK population by ethnicity – regional ethnic diversity (22nd Dec **2022**). *Retrieved from* https://www.ethnicity-facts-figures.service.gov.uk/uk-population-by-ethnicity/national-and-regional-populations/regional-ethnic-diversity/latest/ (last accessed on 11th Sep 2025).
8. Spence JD and Rayner BL. Hypertension in Blacks: individualized therapy based on renin/aldosterone phenotyping. *Hypertension*. **2018**, *72(2)*: 263–269.
9. Kaalep A, Sera T, Rijnsdorp S *et al*. Feasibility of state-of-the-art PET/CT systems performance harmonization. *Eur J Nucl Med Mol Imaging* **2018**, *45*: 1344–1361.
10. Kaalep A, Burggraaff CN, Pieplenbosch S *et al*. Quantitative implications of the updated EARL 2019 PET–CT performance standards. *EJNMMI Phys.* **2019**, *6*: 28.
11. Schneider CA, Rasband WS and Eliceiri KW. NIH Image to ImageJ: 25 years of image analysis. *Nature Methods* **2012**, *9*: 671–675.
12. Schmied C, Nelson MS, Avilov S *et al*. Community-developed checklists for publishing images and image analyses. *Nature Methods* **2024**, *21*: 170–181.

13. Turcu AF, Nhan W, Grigoryan S *et al*. Primary aldosteronism screening rates differ with sex, race, and comorbidities. *J Am Heart Assoc*. **2022**, *11(14)*: e025952.
14. Hundemer GL, Kline GA and Leung AA. How common is primary aldosteronism? *Curr Opin Nephrol Hypertens*. **2021**, *30(3)*: 353–360.
15. Calhoun DA, Nishizaka MK, Zaman MA *et al*. Hyperaldosteronism among black and white subjects with resistant hypertension. Hypertension **2002**, *40(6)*: 892–896.
16. Nanba K and Rainey WE. Genetics in endocrinology: impact of race and sex on genetic causes of aldosterone-producing adenomas. *Eur J Endocrinol*. **2021**, *185(1)*: R1–R11.
